# New chlamydosporol derivatives from the endophytic fungus *Pleosporales* sp. Sigrf05 and their cytotoxic and antimicrobial activities

**DOI:** 10.1038/s41598-020-65148-0

**Published:** 2020-05-18

**Authors:** Daowan Lai, Ziling Mao, Zhiyao Zhou, Siji Zhao, Mengyao Xue, Jungui Dai, Ligang Zhou, Dianpeng Li

**Affiliations:** 1 Department of Plant Pathology, College of Plant Protection, China Agricultural University, Beijing, 100193 China; 2 State Key Laboratory of Bioactive Substance and Function of Natural Medicines, Institute of Materia Medica, Chinese Academy of Medical Science & Peking Union Medical College, Beijing, 100050 China; 3 Guangxi Key Laboratory of Functional Phytochemicals Research and Utilization, Guangxi Institute of Botany, Guilin, 541006 China

**Keywords:** Fungal biology, Drug development

## Abstract

Five new chlamydosporol derivatives, named pleospyrones A-E (**1**–**5**), together with one known congener (**6**), were isolated from the culture of the endophytic fungus *Pleosporales* sp. Sigrf05, obtained from the medicinal plant *Siraitia grosvenorii*. The structures of the new compounds were elucidated mainly by analysis of the HRESIMS, and (1D, 2D) NMR data, while ECD and optical rotation calculations were used to assign the absolute configurations. The plausible biosynthetic pathway of these compounds were proposed. The isolated compounds were evaluated for their cytotoxicity, antifungal and antibacterial activities. Compounds **1**, and **4**–**6** were cytotoxic against the tested cancer cells with IC_50_ values of 1.26~47.5 μM. Compounds **1**–**3** showed moderate antifungal activities against *Magnaporthe oryzae*, while compound **5** displayed weak antibacterial activity.

## Introduction

Chlamydosporol, was a bicyclic, ketal-containing α-pyrone, commonly found in *Fusarium* spp^1^. This type of structure typically contained a methoxyl group at C-4 and a methyl propyl moiety at C-6 of the α-pyrone ring, while C-5 was substituted by a one-carbon substituent such as hydroxymethyl. The chlamydosporol derivatives have also been reported from the other fungi, such as *Tolypocladium inflatum*^2^, *Annulatascus triseptatus*^3^ and *Chaetomium cupreum*^4^, and found to display antibacterial activity^3^ and cytotoxicity^4,5^.

Endophytic fungi are intriguing producers of new bioactive natural products that have potential to be developed as pharmaceuticals or agro-chemicals^6–9^. As a continuation of our interesting in searching for new bioactive compounds from fungal endophytes^10–13^, the fungus Sigrf05 isolated from the medicinal plant *Siraitia grosvenorii*, caught our attention, as the fungal culture showed interesting cytotoxicity in a preliminary assay. This fungus belongs to the order of *Pleosporales*, from which different types of metabolites have been reported, such as diketopiperazines, phthalides^14^, nonadride derivatives^15^, phenyl derivatives^16^, and heptaketides^17^, and displayed cytotoxic, and antifungal activities^14,15,17^.

**Figure 1:**
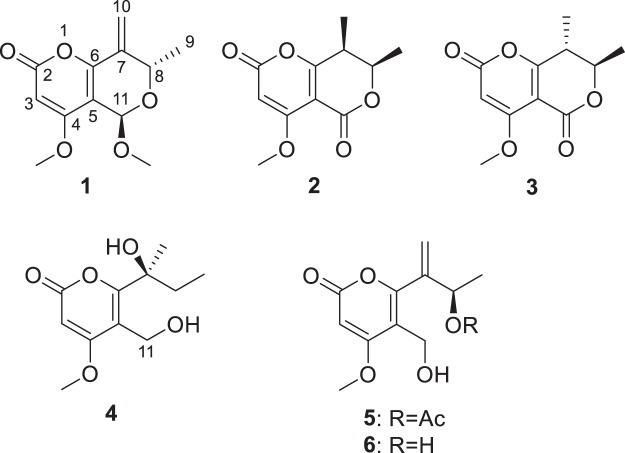
Structures of the isolated compounds (**1**–**6**).

A large-scale fermentation of the titled fungus led to the isolation of six chlamydosporol derivatives, including the new compounds, pleospyrones A-E (**1**–**5**), and one known congener, clearanol A (**6**)^18^ (Fig. 1). Herein, we reported the isolation, structure elucidation, and bioactivities of these compounds. Their biosynthetic pathways were also discussed.

## Results and Discussion

### Structural elucidation

Pleospyrone A (**1**) was isolated as a colorless amorphous solid. It exhibited a prominent pseudomolecular peak at *m*/*z* 239.0914 [M + H]^+^ in the HRESIMS spectrum, indicating a molecular formula of C_12_H_14_O_5_ with six degrees of unsaturation. The ^13^C NMR spectrum (Table 1) displayed 12 carbon resonances, which could be assigned to seven sp^2^-hybridized ones (including one carbonyl group, and six olefinic carbons), two oxygenated methines (δ_C_ 94.8, 65.7), two methoxyl groups (δ_C_ 57.5, 56.0), and one methyl group (δ_C_ 18.1), by the aid of HMQC experiment. Inspection of the ^1^H NMR spectrum (Table 1), revealed the presence of three olefinic protons at δ_H_ 5.66 (s), 5.95 (br. s), 5.47 (br. s), respectively, with the latter two protons attached to one carbon (δ_C_ 114.6, CH_2_), as indicated by the HMQC spectrum. In addition, one dioxygenated methine (δ_H_ 5.38, s), one oxygenated methine appeared as a quartet (δ_H_ 4.76, q) that obviously bonded to a methyl group (δ_H_ 1.47, d), along with two methoxyl groups (δ_H_ 3.90, 3.47) were discerned. The aforementioned functionalities only account for four degrees of unsaturation, meaning that the structure must be bicyclic to fulfill the remaining degrees of unsaturation.

**Table 1:** ^1^H and ^13^C NMR Data of **1**–**5**.

Position	1 ^*a*^		2 ^*b*^		3 ^*b*^		4 ^*b*^		5 ^*b*^	
	*δ*_C_, type	*δ*_H_, mult. (*J*)	*δ*_C_, type	*δ*_H_, mult. (*J*)	*δ*_C_, type	*δ*_H_, mult. (*J*)	*δ*_C_, type	*δ*_H_, mult. (*J*)	*δ*_C_, type)	*δ*_H_, mult. (*J*)
2	165.1, C		161.0, C		160.9, C		163.4, C		162.9, C	
3	90.3, CH	5.66, s	88.8, CH	5.53, s	88.6, CH	5.53, s	88.0, CH	5.48, s	89.3, CH	5.53, s
4	170.4, C		168.7, C		168.7, C		170.5, C		170.1, C	
5	109.9, C		101.0, C		101.6, C		112.0, C		112.5, C	
6	154.0, C		175.4, C		173.0, C		167.0, C		160.1, C	
7	138.3, C		37.0, CH	2.76, qd (7.2, 3.0)	38.4, CH	2.86, dq (9.1, 7.0)	77.4, C	2.06, dq (14.6, 7.4)	140.6, C	
8	65.7, CH	4.76, q (6.3)	73.9, CH	4.69, qd (6.5, 3.0)	77.2, CH	4.32, dq (9.1, 6.3)	34.8, CH_2_	1.75, dq (14.6, 7.4)	70.3, CH	5.47, q (6.6)
9	18.1, CH_3_	1.47, d (6.3)	16.6, CH_3_	1.43, d (6.5)	19.1, CH_3_	1.51, d (6.3)	8.1, CH_3_	0.91, t (7.4)	20.2, CH_3_	1.45, d (6.6)
10	114.6, CH_2_	5.95, br. s; 5.47, br. S	10.4, CH_3_	1.31, d (7.2)	12.7, CH_3_	1.37, d (7.0)	28.2, CH_3_	1.58, s	120.9, CH_2_	5.67, s 5.55, s
11	94.8, CH	5.38, s	159.4, C		158.9, C		53.1, CH_2_	4.75, d (12.2); 4.70, d (12.2)	55.7, CH_2_	4.42, d (12.2) 4.37, d (12.2)
4-OCH_3_	57.5, CH_3_	3.90, s	56.9, CH_3_	3.94, s	56.9, CH_3_	3.94, s	56.5, CH_3_	3.87, s	56.4, CH_3_	3.88, s
11-OCH_3_	56.0, CH_3_	3.47, s								
8-OAc									170.3, C 21.0, CH_3_	2.04, s

^*a*^Recorded in CD_3_OD.

^*b*^Recorded in CDCl_3_.

**Figure 2:**
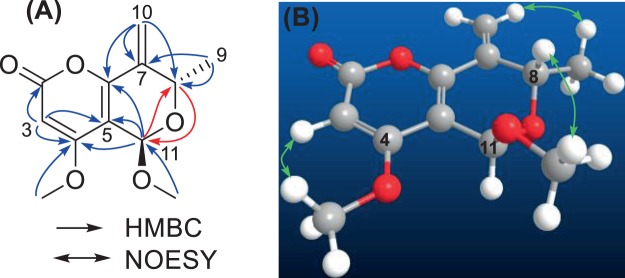
Selected HMBC (**A**) and NOESY (**B**) correlations of **1**.

The planar structure of **1** was established by analysis of the HMBC spectrum (Fig. 2A). A 2-pyrone moiety was constructed firstly, as correlations were seen from H-3 (δ_H_ 5.66, s) to C-2 (δ_C_ 165.1), C-4 (δ_C_ 170.4), and C-5 (δ_C_ 109.9), from H-11 (δ_H_ 5.38, s) to C-4, C-5, and C-6 (δ_C_ 154.0), as well as from 4-OMe (δ_H_ 3.90, s) to C-4. The correlations from H_2_-10 (δ_H_ 5.95, 5.47) to C-6, C-7 (δ_C_ 138.3), and C-8 (δ_C_ 65.7) were used to piece the exocyclic double bond and the pyrone ring together, while correlations from terminal methyl group (CH_3_-9, δ_H_ 1.47, d) to C-7 and C-8, further extend the above moiety to C-9. Another methoxyl group (δ_H_ 3.47, s; δ_C_ 56.0) was positioned at C-11 as it showed correlation to C-11 (δ_C_ 94.8). Additional key correlations of H-11/C-8, and H-8 (δ_H_ 4.76, q)/C-11, were used to establish the gross structure of **1** as shown in Fig. 1. This was corroborated by the NOESY correlations of H-3/4-OMe, and H-10b (δ_H_ 5.47, br. s)/CH_3_-9. The correlation between H-8 and 11-OMe (δ_H_ 3.47, s) indicated that these protons were directed to a same face (Fig. 2B). Thus, the relative configuration of **1** was established.

**Figure 3:**
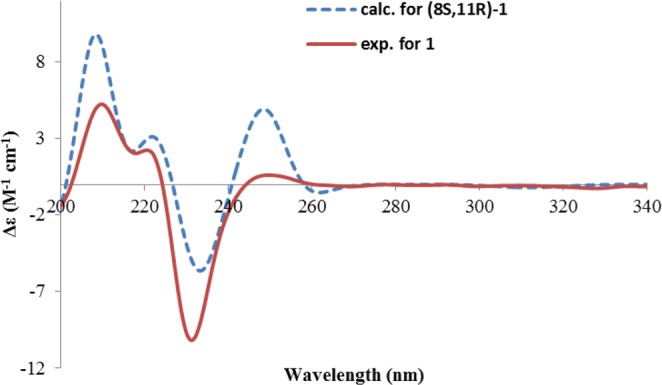
Calculated and experimental ECD spectra of **1**.

The absolute configuration was determined by TDDFT ECD calculation, which was a powerful tool in solving the stereochemistry of complex natural products^19^. A randomly selected structure of **1** (8 *S*, 11 *R*) was subjected to conformational search using the Merck Molecular Force Field (MMFF), followed by geometry optimization at B3LYP/6-31 G (d) level, which resulted in only one predominant conformer (Fig. 2B). In this conformer, the dihydropyran ring adopted a half-chair conformation, in which H-8 was pseudo-axial, while H-11 was pseudo-equatorial. Subsequent TDDFT ECD calculation of this conformer was carried out at PBE0/TZVP level with the PCM solvent model in MeOH. The measured CD spectrum of **1** displayed strong positive Cotton effects at 210 and 220 nm, and a weak positive at 250 nm, while a strong negative peak at 231 nm, reflecting a strong interaction between the intrinsic chromophores. The calculated spectrum reproduced well these peaks as shown in Fig. 3, therefore, the absolute configuration of pleospyrone A (**1**) was established as 8 *S*, 11 *R*.

**Figure 4:**
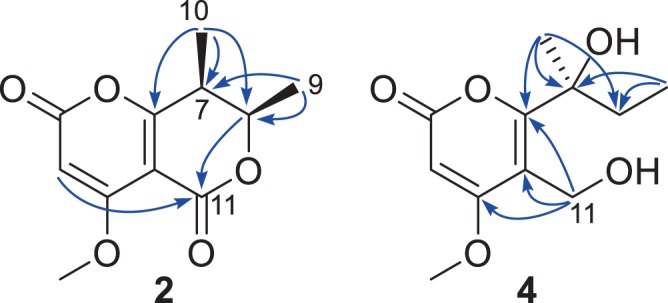
Selected HMBC correlations of **2** and **4**.

**Figure 5:**
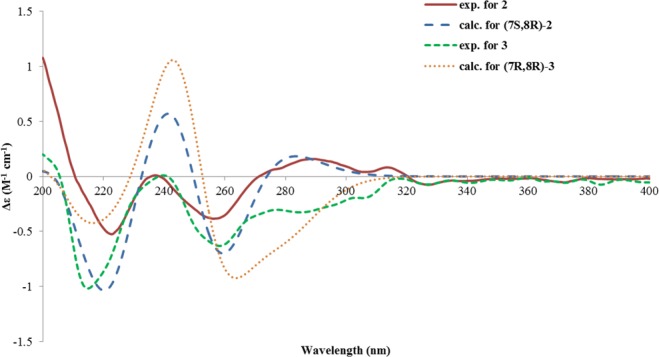
Calculated and experimental ECD spectra for **2** and **3**.

Pleospyrone B (**2**) was isolated as a pale-yellow oil. Its molecular formula was determined as C_11_H_12_O_5_ by HRESIMS. The NMR data of **2** were similar to those of **1** (Table 1), indicating it to be a 2-pyrone derivative. Detailed comparison of the data revealed that the exocyclic double bond of **1** was missing in **2**, instead, one methyl group (δ_H_ 1.31, d; δ_C_ 10.4, CH_3_-10) and a methine group (δ_H_ 2.76, qd; δ_C_ 37.0, C-7) were present, suggesting that the C-7/C-10 double bond was reduced in **2**. In addition, the acetal group in **1** was replaced by an ester group (δ_C_ 159.4, C-11) in **2**, which could explain the significant downfield shift of C-6 (+21.4 ppm), while C-5 was upfield shifted (-8.9 ppm). This was proved by analysis of the HMBC spectrum, in which correlations from CH_3_-10 to C-6 (δ_C_ 175.4), C-7, and C-8 (δ_C_ 73.9), from H_3_-9 (δ_H_ 1.43, d) to C-7, and C-8, from H-8 (δ_H_ 4.69, qd) to C-11, and long-range correlation from H-3 (δ_H_ 5.53, s) to C-11 were observed (Fig. 4). The small coupling constant (3.0 Hz) between H-7 and H-8 indicated the *cis* relationship for both protons, which was similar to that of inflatin C^2^. The absolute configuration of **2** was also assigned by quantum chemical ECD calculations. A randomly selected configuration of **2** (7 *R*, 8 *S*) was used for the calculation. Geometry optimization of the MMFF conformers at B3LYP/6-31 G(d) *in vacuo* resulted in two major conformers (**2a**, 94.6%, and **2b**, 5.3%, see Supplementary Fig. S1), in which one methyl was pseudo-equatorial, while the other was pseudo-axial with regard to the half-chair conformation of the 2-dihydropyrone ring. Subsequent TDTFT ECD calculations of both conformers at the CAMB3LYP/TZVP (PCM = MeOH) level gave an almost opposite ECD spectrum except in the long-wavelength region (ca. 273~310 nm, Supplementary Fig. S2), and the resulting Boltzmann-averaged spectrum was opposite to the experimental spectrum of **2** (Supplementary Fig. S2). On the contrary, the spectrum for (7 *S*, 8 *R*)-**2** matched well the experimental one (Fig. 5). Therefore, the absolute configuration of **2** was assigned as 7 *S*, 8 *R*.

Pleospyrone C (**3**) was isolated as an isomer of **2**, and both have a same molecular formula. Inspection of the NMR data (Table 1) revealed their great similarities, and the differences were only ascribed to the different orientation of the methyl groups. The large coupling constant (9.1 Hz) between H-7 and H-8 was reminiscent of their *trans* relationship, similar to that of isochlamydosporol^2^. With this relative configuration in hand, the absolute configuration of **3** was then assigned by comparing the calculated and experimental ECD spectra. An arbitrary input structure of **3** (7 *S*, 8 *S*) was subjected to conformational search and geometry optimization at the B3LYP/6-31 G(d) level, which resulted in two predominant conformers (**3a**, 24.7%, and **3b**, 75.2%, see Supplementary Fig. S3), where both methyl groups were pseudo-equatorial in **3b**, while pseudo-axial in **3a**. These two conformers also give an opposite spectrum except in the high wavelength range (ca. 277~310 nm, Supplementary Fig. S4). The Boltzmann-averaged spectrum of (7 *S*, 8 *S*)-**3** gave an opposite image to the recorded spectrum of **3** (Supplementary Fig. S4). Then, the absolute configuration of **3** was determined to be 7 *R*, 8 *R* (Fig. 5) by comparing the spectrum of (7 *R*, 8 *R*)-**3** with the experimental spectrum. Compound **3** was thus determined to be a 7-epimer of **2**. Interestingly, their ECD spectra were quite similar, while only opposite in the long-wavelength range (ca.270–320 nm) (Fig. 5). Such differences were attributed to a change in the orientation of CH_3_-10 (axial in **2a**, vs equatorial in **3b**).

**Figure 6:**
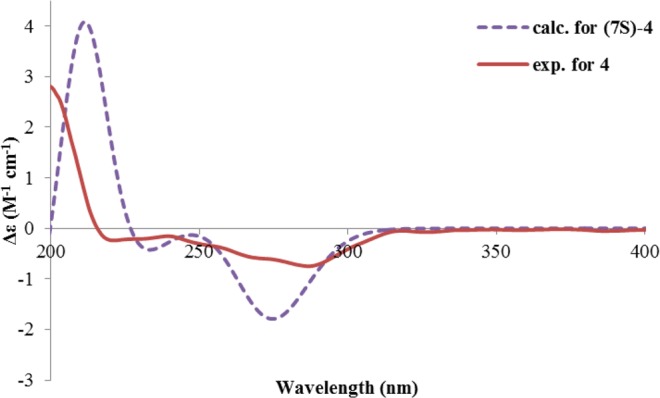
Calculated and experimental ECD spectra of **4**.

Pleospyrone D (**4**) had a molecular formula of C_11_H_16_O_5_, as determined by HRESIMS. It was also an α-pyrone as inferred from its similar NMR data to those of **1**–**3** (Table 1). Analysis of the HMBC spectrum revealed that there was a hydroxymethyl group at C-11, while C-6 was substituted with a 1-hydroxy-1-methyl-propyl group. As correlations could be seen from H_2_-11 (δ_H_ 4.75, 4.70, each d) to C-4 (δ_C_ 170.5,), C-5 (δ_C_ 112.0), and C-6 (δ_C_ 167.0), from CH_3_-10 (δ_H_ 1.58, s) to C-6, C-7 (δ_C_ 77.4), and C-8 (δ_C_ 34.8), as well as from CH_3_-9 (δ_H_ 0.91, t) to C-7, and C-8 (Fig. 4). The absolute configuration of **4** was established also by ECD computations. The calculated spectra at the CAMB3LYP/TZVP (PCM = MeOH) level gave a satisfactory fit with the experimental data (Fig. 6), suggesting a 7 *S* configuration of **4**. In addition, the optical rotation of (7 *S*)*-***4** in methanol was calculated at the b3lyp/6–31 + g(d,p) level, which also match the experimental value (calcd. [α]_D_ -53.6; exp. [α]^24^_D_ -21.8 (*c* 0.50, MeOH)).

Pleospyrone E (**5**) was determined to be the 8-acetyl derivative of the co-isolated clearanol A (**6**)^18^, by comparison of their NMR data and analysis of the HMBC spectrum. The key HMBC correlation from H-8 (δ_H_ 5.47, q) to the carbonyl (δ_C_ 170.3) of the acetyl group was seen. The absolute configuration of **5** was determined to be the same as that of clearanol A (**6**) by the observation of a similar CD profile between both compounds (Supplementary Fig. S6). Clearanol A was previously isolated from the fungus derived from a microbial mat collected from an iron-rich natural spring^18^.

**Figure 7:**
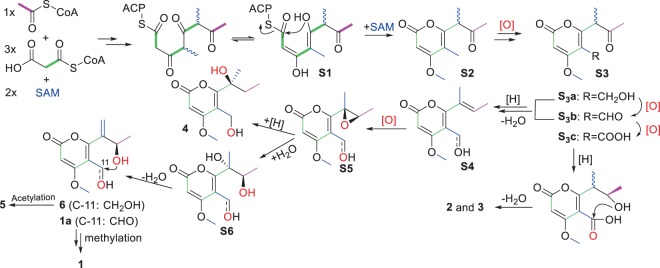
Hypothetical biosynthetic pathways of **1**–**6**.

In this study, six polyketides (**1**–**6**) with an α-pyrone motif were isolated, and their plausible biosynthetic pathways were proposed (Fig. 7). The PKS could incorporate one acetyl CoA, three malonyl CoA, and two *S*-adenosyl methionines (SAM) to build up the tetraketide (**S1**), which was released by esterification to give the α-pyrone, followed by *O*-methylation at C-4 to produce **S2**. Oxidation at C-11 should give the oxygenated derivatives (**S3**), and reduction of the 8-keto group would give the 8-hydroxy derivatives. Lactonization of the 8-hydroxylated **S**_**3**_**c** should give the lactone-pyrones (**2** and **3**), while dehydration of the 8-hydroxylated **S**_**3**_**a** and **S**_**3**_**b** would give the Δ^7^ derivatives (**S4**), which could be epoxidized to produce the epoxides **S5**. Reduction of the epoxide would give **4**, while the nucleophilic attack of H_2_O to the epoxy ring could produce the vicinal diols **S6**, which were then dehydrated to give **6** and **1a**. Further acetylation of **6** should give rise to **5**. The attack from 8-OH to the aldehyde group in **1a**, followed by *O*-methylation could produce **1**.

### Cytotoxic and antimicrobial activities

The six isolated α-pyrone derivatives (**1**–**6**) were structurally related to chlamydosporol, a mycotoxin widely occurred in *Fusarium* spp^1^., and they differed only in the eastern part (i.e. non-α-pyrone part). It was found that chlamydosporol displayed cytotoxicity to mouse and human fibroblast cells, caused feed refusal and weight loss of rats, and mortality to chick embryos^5^, and also exhibited toxicity to *Artemia salina* larvae^20^. Hence, the cytotoxicities of compounds **1**–**6** were also evaluated (Table 2). Compounds **1**, and **4**–**6** were active against the tested cancer cell lines including HCT-116, HepG2, BGC-823, NCI-H1650 and Daoy, with IC_50_ values in the range of 1.26~47.5 μM, though not as active as the positive control (taxol), while **2** and **3** were inactive (IC_50_ > 50 μM). Among them, compound **5** was active against all the five tested cell lines, with IC_50_s of 1.17~20.7 μM, compound **1** was effective against all the cell lines except HCT-116 (IC_50s_ 1.26~15.1 μM), whereas compounds **4** and **6** were selectively cytotoxic against NCI-H1650 cells with IC_50_ values of 29.6, and 47.5 μM, respectively. It seemed that the substituents at C-7 had strong influence to the activity, when C-7 was a methine as in **2** and **3**, the compounds were inactive, while when it was a quaternary carbon, as in **1**, and **4**–**6**, the cytotoxicity was increased. In addition, the most potent compounds (i.e, **1** and **5**) all had a C7/C10 exocyclic double bond. However, the loss of one acetyl group, as in the case of **6** vs **5**, the activity was significantly decreased, meaning that a suitable lipophilicity was also important for the cytotoxicity. More structures should be evaluated to get a better understanding of the structure-activity-relationship. And the mechanism of action towards these cells is yet to be revealed.

**Table 2:** Cytotoxicities of the Isolated Compounds (IC_50_, μM).

Compound	HCT-116	HepG2	BGC-823	NCI-H1650	Daoy
1	>50.0	5.07	1.26	15.1	2.72
2	>50.0	>50.0	>50.0	>50.0	>50.0
3	>50.0	>50.0	>50.0	>50.0	>50.0
4	>50.0	>50.0	>50.0	29.6	>50.0
5	1.17	20.3	20.7	6.26	19.9
6	>50.0	>50.0	>50.0	47.5	>50.0
Taxol^*a*^	1.90 × 10^−3^	1.46 × 10^−2^	1.07 × 10^−4^	1.10	5.04 × 10^−3^

^*a*^Positive control.

The isolated compounds were further evaluated for their antifungal activities (Table 3). Among them, compounds **1**–**3** showed moderate inhibition against the spore germination of the rice blast fungus *Magnaporthe oryzae* with IC_50_ value of 98.73, 47.77, and 51.08 µg/mL, respectively, while the other compounds did not display any significant activity when tested at a concentration of 200 µg/mL. It was interesting that all the active compounds were bicyclic, whereas the monocyclic compounds (i.e., **4**–**6**) were inactive. More derivatives should be evaluated to get a better structure-activity-relationship.

**Table 3:** Inhibitory Activity against the Spore Germination of *M. oryzae*.

Compound^*a*^	IC_50_ (µg/mL)
**1**	98.73
**2**	47.77
**3**	51.08
Carbendazim^*b*^	8.70

^*a*^The other compounds were inactive (IC_50_ > 200 µg/mL).

^*b*^Positive control.

The isolated compounds were also screened for their antibacterial activities. Among them, compound **5** exhibited a weak inhibition against *Bacillus subtilis*, *Agrobacterium tumefaciens*, *Ralstonia solanacearum*, and *Xanthomonas vesicatoria* with the same MIC value of 100.0 µM, however, the other tested compounds were inactive (MICs> 125 μM).

## Conclusion

Six chlamydosporol congeners, including five new pleospyrones A-E (**1**–**5**), and the known clearanol A (**6**) were isolated from the endophytic fungus *Pleosporales* sp. Sigrf05 that resided inside the medicinal plant *Siraitia grosvenorii*. The absolute configuration of the new compounds was unambiguously determined by applying the quantum chemical calculations (ECD and optical rotation). The occurrence of these polyketides added a new diversity to the α-pyrone class of natural products, and also reflected the presence of abundant post-PKS tailoring enzymes in their biosynthesis, which merited further study. Compounds **1**, and **4**–**6** were cytotoxic, while compounds **1**–**3** displayed moderate antifungal activities. A preliminary structure-activity-relationship analysis revealed that the substituents at C-7 and the lipophilicity were relevant to the cytotoxicity, while bicyclic structures were important for the antifungal activities. These polyketides could be interesting candidates for the development of cytotoxic and antimicrobial agents.

## Methods

### General experimental procedures

The optical rotations were measured on a Rudolph Autopol IV automatic polarimeter (Rudolph Research Analytical, New Jersey, USA). The ultraviolet (UV) spectra were scanned by a TU-1810 UV-VIS spectrophotometer (Beijing Persee General Instrument Co., Ltd., Beijing, China). Circular dichroism (CD) spectra were recorded on a JASCO J-810 CD spectrometer (JASCO Corp., Tokyo, Japan). High resolution electrospray ionization mass spectrometry (HRESIMS) spectra were recorded on an LC1260-Q-TOF/MS 6520 machine (Agilent Technologies, CA, USA). ^1^H, ^13^C, and 2D NMR spectra were measured on an Avance 400 NMR spectrometer (Bruker BioSpin, Zurich, Switzerland). Chemical shifts were expressed in *δ* (ppm) referenced to the inner standard TMS (for CDCl_3_), or the solvent residual peaks (*δ*_H_ 3.31/*δ*_C_ 49.0 for CD_3_OD), and coupling constants (*J*) in hertz. Sephadex LH-20 (Pharmacia Biotech, Uppsala, Sweden), and silica gel (200~300 mesh, Qingdao Marine Chemical Inc., Qingdao, China) were used for column chromatography. Semi-preparative HPLC separation was carried out on a Lumtech K-501 pump (Lumiere Tech. Ltd., Beijing, China) with a K-2501 UV detector using a Luna-C18 column (250 mm × 10 mm i.d., 5 μm, Phenomenex Inc., Torrance, CA, USA). High performance liquid chromatography (HPLC) analysis was performed using a Shimadzu LC-20A instrument with a SPD-M20A photodiode array detector (Shimadzu Corp., Tokyo, Japan) and an analytic C_18_ column (250 mm × 4.6 mm i.d., 5 µm; Phenomenex Inc., Torrance, California, USA). The precoated silica gel GF-254 plates (Qingdao Marine Chemical Inc., China) on glass were used for analytical thin layer chromatography (TLC). Spots were visualized under UV light (254 or 356 nm) or by spraying with 10% H_2_SO_4_ in 95% ethanol followed by heating.

### Fungal material and fermentation

The fungus (strain No. Sigrf05) was isolated from the healthy tuberous roots of the medicinal plant *Siraitia grosvenorii*, which was collected from Guangxi Province of China in June 2015. The internal transcribed spacer (ITS) sequence of the rDNA gene was sequenced and uploaded to the NCBI GenBank with the accession No. KT369815. BLAST analysis of the sequence revealed that this fungus belonged to the order of *Pleosporales*, as it showed 99.83% of identity to two unclassified species within this order (GenBank accession Nos. HQ832808.1 and JQ809679.1) (Table S1). A voucher specimen was deposited in the Department of Plant Pathology, China Agricultural University.

The fungus was grown on potato dextrose agar (PDA) plates at 25 °C for 8~10 days. Then, four to five plugs of agar medium (0.5 cm × 0.5 cm) with fungal hyphae were transferred to several 250 mL Erlenmeyer flasks each containing 100 mL potato dextrose broth (PDB) medium to prepare the seed culture, and incubated on a rotary shaker at 150 rpm and 25 °C for 7 days. The scale-up fermentation was carried out in 1 L- Erlenmeyer flasks, each containing 150 g rice and 150 mL distilled water. Each flask was inoculated with a seed culture. The static fermentation was kept at 25 °C for approximately 45 days before harvest.

### Extraction and isolation

The fermented rice substrates in 25 flasks were combined, and extracted with MeOH by exhaustive maceration (3 × 10 L) at room temperature. After filtration, the filtrate was concentrated under vacuum at 40 °C. The brown residue was suspended in water and sequentially partitioned with petroleum ether, EtOAc, and *n*-BuOH to give their corresponding fractions. Then, the EtOAc extracts were combined and concentrated under vacuum at 40 °C to obtain a brownish residue (40.8 g).

The EtOAc extract was subjected to column chromatography (CC) over silica gel (200~300 mesh) eluting with a gradient of petroleum ether−EtOAc (100:0–0:100) to obtain four fractions (fractions A~D).

Fraction B (8.5 g) was separated by Sephadex LH-20 CC using CHCl_3_–MeOH (1:1) as eluent to yield four subfractions (B1~B4). Subfraction B2 and B3 were purified by semi-preparative HPLC using MeOH–H_2_O (60:40) as the mobile phase to afford compound **1** (17.0 mg).

Fraction C (12.0 g) was fractionated by vacuum liquid chromatography (VLC) on silica gel, eluting with a gradient of petroleum ether–EtOAc (100:0–0:100) to yield eight subfractions (C1~C8). Subfraction C4 was subjected to CC over Sephadex LH-20 using CHCl_3_–MeOH (1:1) as eluent, followed by purification using semi-preparative HPLC (55% MeOH/H_2_O) to afford compounds **2** (3.6 mg) and **3** (3.8 mg), respectively. Subfraction C7 was separated by semi-preparative HPLC using MeOH–H_2_O (50:50) as eluent to afford compound **5** (10.0 mg). Likewise, compound **4** (9.0 mg) was isolated from subfraction C8 by semi-preparative HPLC (MeOH–H_2_O, 40:60).

Fraction D (5.5 g) was processed in the same manner as that of subfraction C4 using MeOH–H_2_O (38:62) as eluent to afford compound **6** (4.5 mg).

Pleospyrone A (**1**). Colorless amorphous solid; [α]^24^_D_ + 43.5 (*c* 0.40, MeOH); UV (MeOH) λ_max_ (log *ε*) 221 (4.37), 311 (3.70) nm; ECD (*c* = 1.05 × 10^−3^ M, MeOH) λ (Δε) 210 (+5.23), 218 (+2.02), 220 (+2.22), 231 (−10.17), 250 (+0.61) nm; HRESIMS *m*/*z* 239.0914 [M + H]^+^ (calcd for C_12_H_15_O_5_, 239.0914); ^1^H (CD_3_OD, 400 MHz) and ^13^C (CD_3_OD, 100 MHz) NMR data, see Table 1.

Pleospyrone B (**2**). Pale-yellow oil; [α]^24^_D_ −11.9 (*c* 0.35, MeOH); UV (MeOH) λ_max_ (log *ε*) 213 (4.12), 249 (3.75), 279 (sh) (3.43) nm; ECD (*c* = 1.12 × 10^−3^ M, MeOH) λ (Δε) 223 (−0.52), 237 (+0.01), 256 (−0.38), 289 (+0.16), 314 (+0.08) nm; HRESIMS *m*/*z* 225.0757 [M + H]^+^ (calcd for C_11_H_13_O_5_, 225.0757); ^1^H (CDCl_3_, 400 MHz) and ^13^C (CDCl_3_, 100 MHz) NMR data, see Table 1.

Pleospyrone C (**3**). Pale-yellow oil; [α]^24^_D_ −19.4 (*c* 0.35, MeOH); UV (MeOH) λ_max_ (log ε) 212 (4.18), 250 (3.83), 279 (sh) (3.50) nm; ECD (*c* = 1.12 × 10^−3^ M, MeOH) λ (Δε) 215 (−1.02), 240 (+0.01), 258 (−0.63), 285 (−0.33) nm; HRESIMS *m*/*z* 225.0758 [M + H]^+^ (calcd for C_11_H_13_O_5_, 225.0757); ^1^H (CDCl_3_, 400 MHz) and ^13^C (CDCl_3_, 100 MHz) NMR data, see Table 1.

Pleospyrone D (**4**). Pale-yellow oil; [α]^24^_D_ −21.8 (*c* 0.50, MeOH); UV (MeOH) λ_max_ (log ε) 207 (4.32), 282 (3.73) nm; ECD (*c* = 1.10 × 10^−3^ M, MeOH) λ (Δε) 221 (−0.24), 239 (−0.15), 287 (−0.75), 318 (−0.05) nm; HRESIMS *m*/*z* 229.1065 [M + H]^+^ (calcd for C_11_H_17_O_5_, 229.1071); ^1^H (CDCl_3_, 400 MHz) and ^13^C (CDCl_3_, 100 MHz) NMR data, see Table 1.

Pleospyrone E (**5**). Colorless amorphous solid; [α]^24^_D_ + 2.1 (*c* 0.50, MeOH); UV (MeOH) λ_max_ (log ε) 206 (4.17), 288 (3.78) nm; ECD (*c* = 9.33 × 10^−4^ M, MeOH) λ (Δε) 204 (+1.13), 209 (+1.01), 217 (+1.32), 235 (−1.32), 261 (−0.37), 296 (−0.76) nm; HRESIMS *m*/*z* 291.0842 [M + Na]^+^ (calcd for C_13_H_16_O_6_Na, 291.0839); ^1^H (CDCl_3_, 400 MHz) and ^13^C (CDCl_3_, 100 MHz) NMR data, see Table 1.

### Antifungal assay

The antifungal activities of **1**–**6** were tested against the rice blast pathogen *M. oryzae* using the spore germination assay^21^. Briefly, the tested compounds were dissolved in 10% aqueous ethanol (25 μL) and mixed with a same volume of spore suspension (2 × 10^6^ spores/mL) that prepared from the 7-day old cultures of *M. oryzae*, on a concave glass slide. Slides containing the spores were incubated in a dark moist chamber at 25 °C for 8 h, then the germination status was inspected under a microscope. 10% ethanol was used as the blank control. Carbendazim was used as the positive control. The percentage (%) of inhibition was determined as [(Gb − Gt)/Gb] × 100, where Gb is the average numbers of the germinated spores in the blank control (n = 3), and Gt is the average numbers of those in the treated sets (n = 3). The half-inhibition concentration (IC_50_) of each sample was then calculated by linear regression.

### Antibacterial assay

The antibacterial activities of **1**–**6** were tested against four pathogenic bacteria including *B. subtilis*, *A. tumefaciens*, *R. solanacearum*, and *X. vesicatoria* using the modified broth micro-dilution-MTT assay as described previously^21^. The bacteria were grown in liquid LB medium overnight at 28 °C, and the diluted bacterial suspension (10^6^ cfu/mL) was used for the assay. Streptomycin sulfate was used as the positive control, which showed minimum inhibition concentration (MIC) of 12.5, 25.0, 12.5, and 25.0 µM, respectively, towards these bacteria.

### Cytotoxic assay

Cytotoxicities of **1**–**6** were tested against five human cancer cell lines including colon cancer cells (HCT-116), liver hepatocellular carcinoma cells (HepG2), gastric cancer cells (BGC-823), nonsmall-cell lung carcinoma cells (NCI-H1650), and medulloblastoma cells (Daoy). These cells were seeded in 96-well plates at 1200 cells/well. After 24 h, the tested compounds were added to the cells. Cell viability was determined after a further treatment of 96 h by using the MTT assay^22^. The UV absorption of each well at 570 nm were read using a plate reader. Compounds dissolved in DMSO (final DMSO concentration of 0.1% in each well), were tested in five concentrations with each concentration tested in triplicate. IC_50_ values were then calculated using Microsoft Excel software. Taxol was used as the positive control.

### ECD calculation

The Molecular Merck force field (MMFF) conformational search, geometry optimization and frequency calculations at the B3LYP/6-31 G(d) level *in vacuo*, and TDDFT ECD calculations of the dominant conformers (>1%) at different levels (PBE0/TZVP and CAMB3LYP/TZVP) with the polarizable continuum model (PCM) for MeOH, were performed as described previously^23^. ECD spectrum of each conformer was plotted with the program SpecDis^24^ using the dipole-length computed rotational strengths with Gauss curve and exponential half-width (*σ*) of 0.16 ev for **1**, and 0.3 eV for **2**–**4**, respectively. The equilibrium population of each conformer at 298.15 K was calculated from its relative Gibbs free energies using Boltzmann statistics. The Boltzmann-averaged ECD spectra for (8 *S*, 11 *R*)-**1**, (7 *R*, 8 *S*)-**2**, (7 *S*, 8 *S*)-**3**, and (7 *S*)-**4** were generated according to the equilibrium population of the lowest energy conformers of each structure, while the calculated spectra for (7 *S*, 8 *R*)-**2** and (7 *R*, 8 *R*)-**3** were obtained by mirroring those of the enantiomers. The calculated spectra were then compared with the experimental spectra to determine the absolute configuration. The calculated ECD spectra were scaled (y-axes) (by 0.5 for **1** and **4**, 0.05 for **2**, and 0.1 for **3**) for a better comparison with the experimental data.

### Optical rotation calculation

The b3lyp/6-31 g(d)-optimized conformers of (7 *S*)-**4** were used to calculate the optical rotations (OR). The OR calculations were carried out using the time-dependent DFT methods at the B3LYP/6-31 + G(d,p) level, with the PCM model for methanol, as described previously^23^. Boltzmann statistics analysis was employed to calculate the overall OR.

## Supplementary information

Supplementary information
