# Supplementary material for: New chlamydosporol derivatives from the endophytic fungus Pleosporales sp. Sigrf05 and their cytotoxic and antimicrobial activities
Source: Sci Rep. 2020 May 18;10:8193. doi: 10.1038/s41598-020-65148-0 (PMC7234987; doi:10.1038/s41598-020-65148-0)
Supplement: Supplementary file 1 — Supplementary information [file 41598_2020_65148_MOESM1_ESM.pdf]

Supplementary File

New chlamydosporol derivatives from the endophytic fungus *Pleosporales* sp. Sigrf05 and their cytotoxic and antimicrobial activities

Daowan Lai[1], Ziling Mao[1], Zhiyao Zhou[1], Siji Zhao[1], Mengyao Xue[1], Jungui Dai[2], Ligang Zhou[1] & Dianpeng Li[3]

[1]Department of Plant Pathology, College of Plant Protection, China Agricultural University, Beijing 100193, China. [2]State Key Laboratory of Bioactive Substance and Function of Natural Medicines, Institute of Materia Medica, Chinese Academy of Medical Science & Peking Union Medical College, Beijing 100050, China. [3]Guangxi Key Laboratory of Functional Phytochemicals Research and Utilization, Guangxi Institute of Botany, Guilin 541006, China.

Daowan Lai and Ziling Mao contributed equally to this work.

Correspondence and requests for materials should be addressed to Ligang Zhou (email: lgzhou@cau.edu.cn) or Dianpeng Li (email: ldp@gxib.cn).

Contents

Table S1. NCBI blastn results for the ITS sequence of Sigrf05 (Accession No. KT369815.1) 3

Figure S1. Optimized geometries of the predominant conformers for (7*R*, 8*S*)-**2** at the B3LYP/6-31G(d) level in the gas phase. 4

Figure S2. Calculated ECD spectra for (7*R*,8*S*)-**2a**, (7*R*,8*S*)-**2b**, and the Boltzmann-averaged spectrum. 4

Figure S3. Optimized geometries of the predominant conformers for (7*S*, 8*S*)-**3** at the B3LYP/6-31G(d) level in the gas phase. 5

Figure S4. Calculated ECD spectra for (7*S*,8*S*)-**3a**, (7*S*,8*S*)-**3b**, and the Boltzmann-averaged spectrum. 5

Figure S5. Optimized geometries of the predominant conformers for **4** at the B3LYP/6-31G(d) level in the gas phase. 6

Figure S6. ECD spectra of **5** and **6**. 6

NMR, HRESIMS, and CD spectra of 1 7

NMR, HRESIMS, and CD spectra of 2. 10

NMR, HRESIMS, and CD spectra of 3. 13

NMR, HRESIMS, and CD spectra of 4. 15

NMR, HRESIMS, and CD spectra of 5. 18

Table S1. NCBI blastn results for the ITS sequence of Sigrf05 (Accession No. KT369815.1)

Description	Max Score	Total Score	Query cover	E Value	Per. Ident	Accession
Pleosporales sp. LH70	1061	1160	99%	0	99.83	HQ832808.1
Pleosporales sp. YF04U3	1061	1160	99%	0	99.83	JQ809679.1
Pleosporales sp. Sigrf03	1050	1149	98%	0	100	KT369813.1
Pleosporales sp. C1	1029	1112	95%	0	99.82	LC168756.1
Pleosporales sp. p47	1014	1095	93%	0	100	LC169795.1
Pleosporales sp. d75	1005	1076	91%	0	100	LC169832.1
Setophoma sp. HD-2014 isolate DO97	1000	1084	97%	0	98.93	KP050652.1
Pleosporales sp. p103	992	992	85%	0	99.82	LC169822.1
Setophoma yingyisheniae CGMCC 3.19527	939	939	80%	0	100	NR_164602.1
Setophoma sp. FL-2019d isolate LF1420	939	939	80%	0	100	MK511912.1
Uncultured fungus clone LX042399-122-056-B07	935	935	91%	0	95.35	GQ999533.1
Setophoma sp. FL-2019d isolate LF556	933	933	80%	0	99.8	MK511942.1
Setophoma sp. FL-2019d isolate LF448	933	933	80%	0	99.8	MK511939.1
Setophoma sp. FL-2019d isolate LF407	933	933	80%	0	99.8	MK511937.1
Setophoma sp. FL-2019d isolate LF341	933	933	80%	0	99.8	MK511929.1
Fungal endophyte isolate 5470	915	915	80%	0	99.02	JQ846051.1

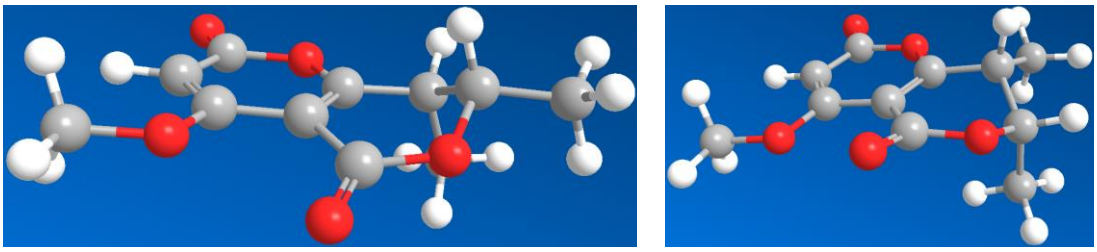

(7*R*, 8*S*)-**2a**, 94.6% (7*R*, 8*S*)-**2b**, 5.3 %

Figure S1. Optimized geometries of the predominant conformers for (7*R*, 8*S*)-**2** at the B3LYP/6-31G(d) level in the gas phase.

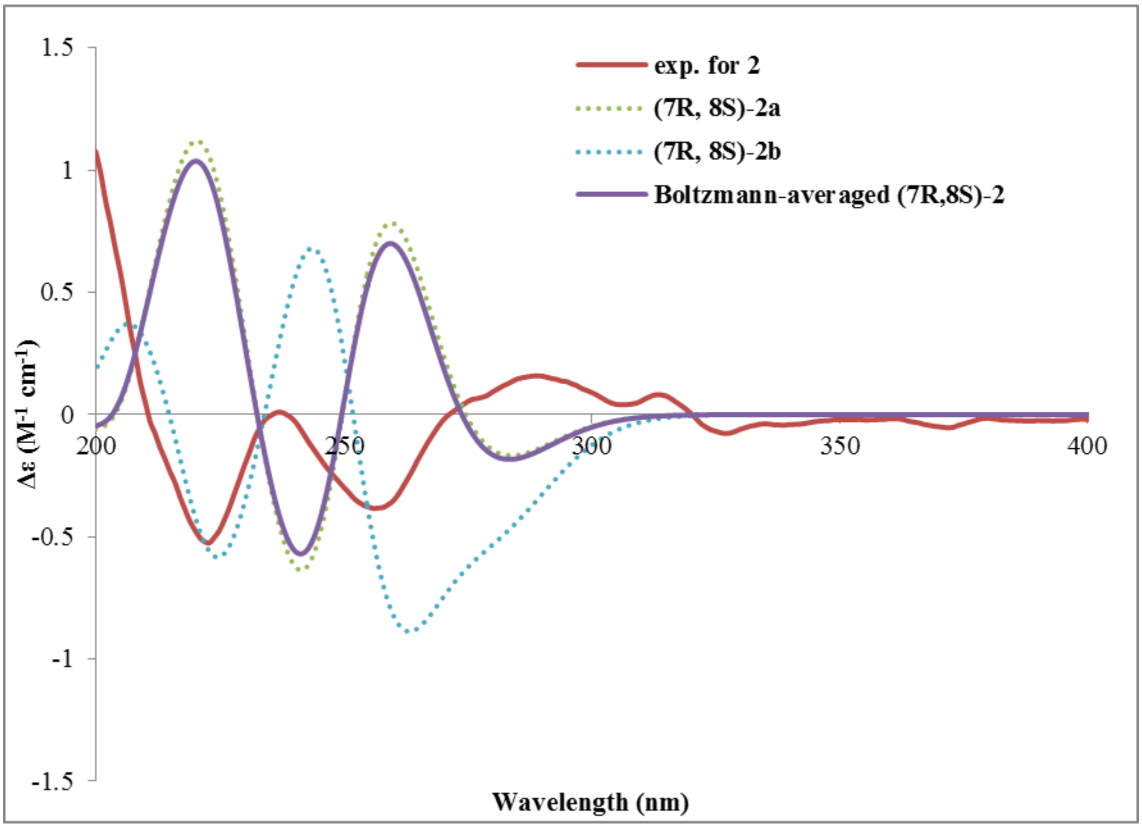

Figure S2. Calculated ECD spectra for (7*R*,8*S*)-**2a**, (7*R*,8*S*)-**2b**, and the Boltzmann-averaged spectrum.

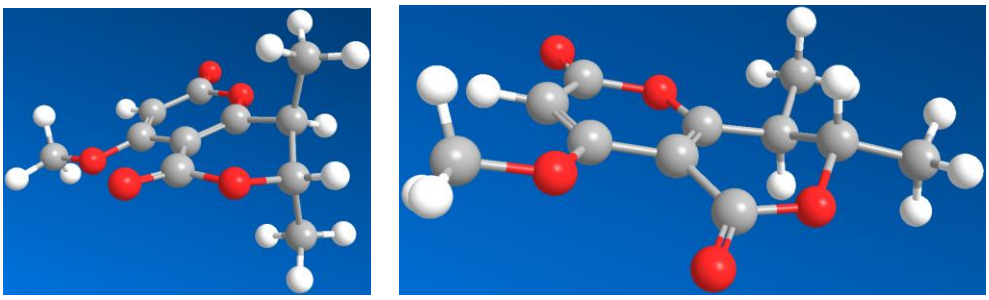

(7*S*, 8*S*)-**3a**, 24.7% (7*S*, 8*S*)-**3b**, 75.2%

Figure S3. Optimized geometries of the predominant conformers for (7*S*, 8*S*)-**3** at the B3LYP/6-31G(d) level in the gas phase.

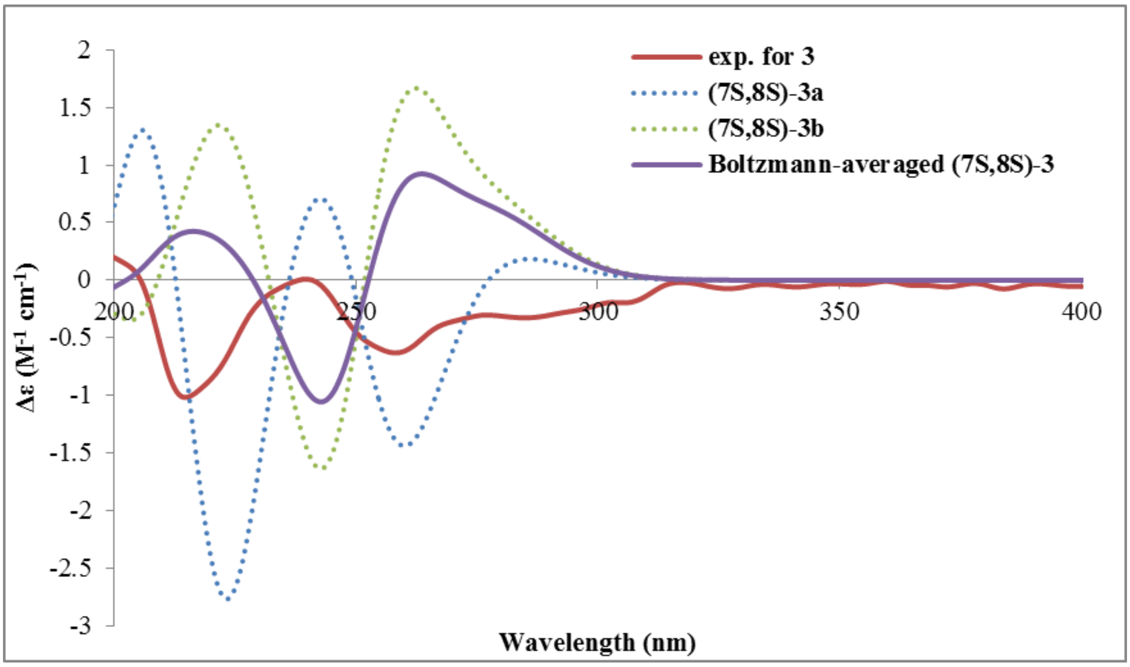

Figure S4. Calculated ECD spectra for (7*S*,8*S*)-**3a**, (7*S*,8*S*)-**3b**, and the Boltzmann-averaged spectrum.

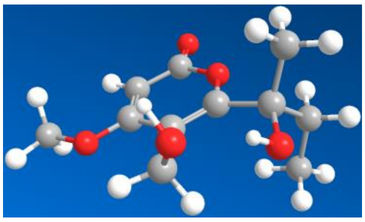

4-c, 34.4%

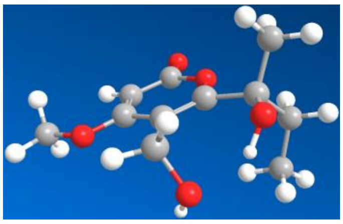

4-e, 20.9%

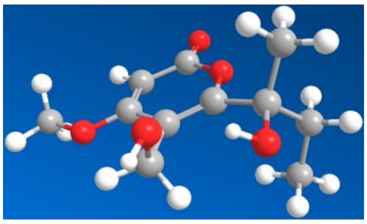

4-f, 11.6%

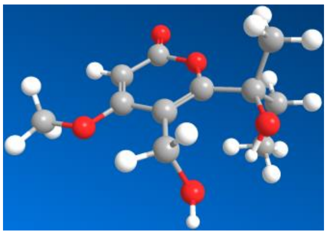

4-g, 4.9%

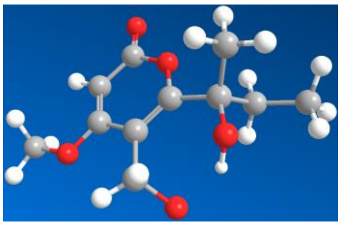

4-i, 10.7%

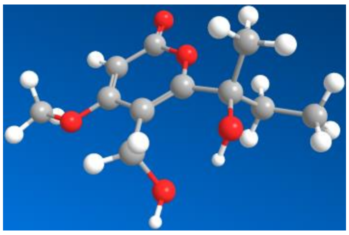

4-j, 3.1%

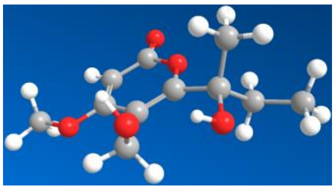

4-k, 8.0%

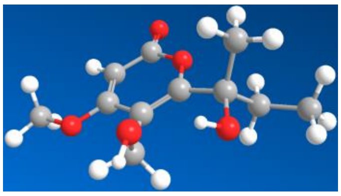

4-r, 2.1%

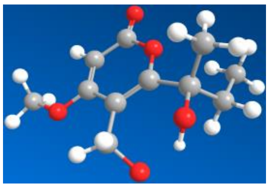

4-s, 1.1%

Figure S5. Optimized geometries of the predominant conformers for **4** at the B3LYP/6-31G(d) level in the gas phase.

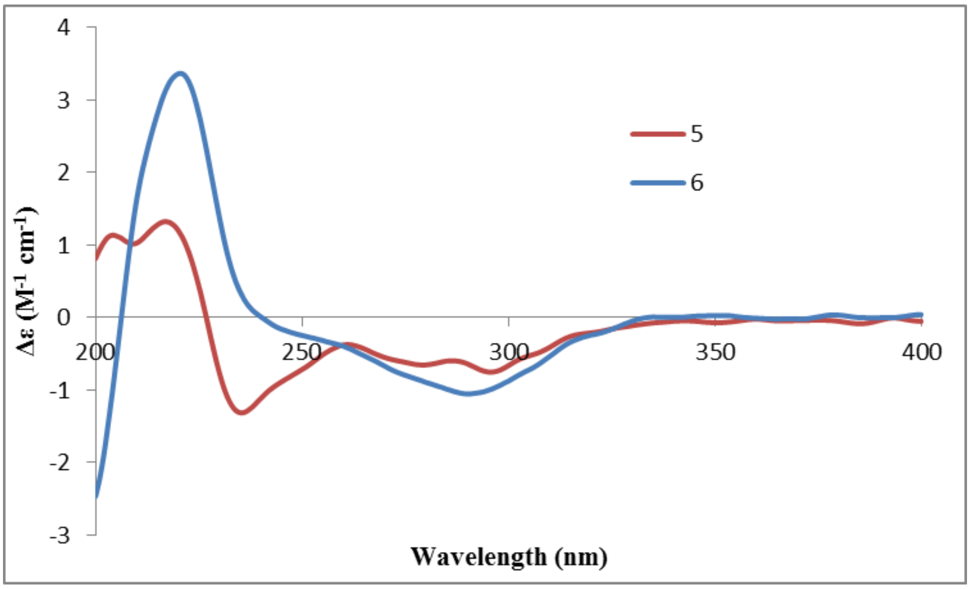

Figure S6. ECD spectra of **5** and **6**.

NMR, HRESIMS, and CD spectra of 1

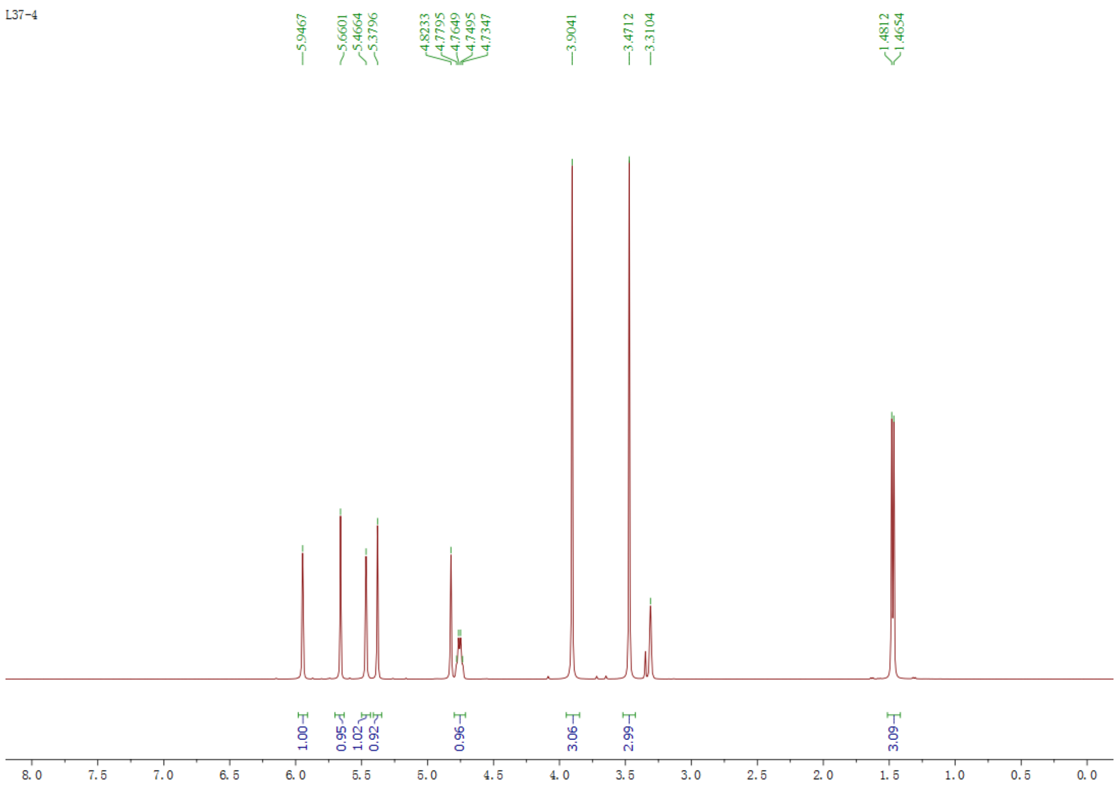

^{1}H NMR spectrum of **1** (CD_3OD, 400MHz).

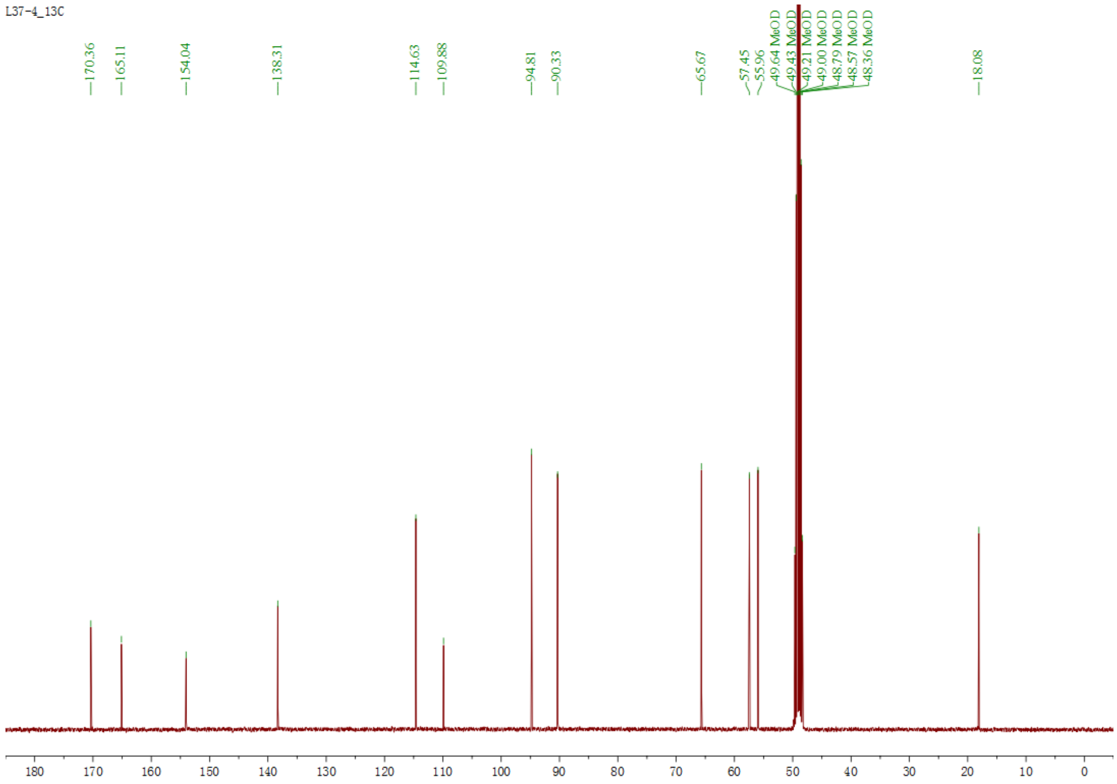

^{13}C NMR spectrum of **1** (CD_3OD, 100MHz).

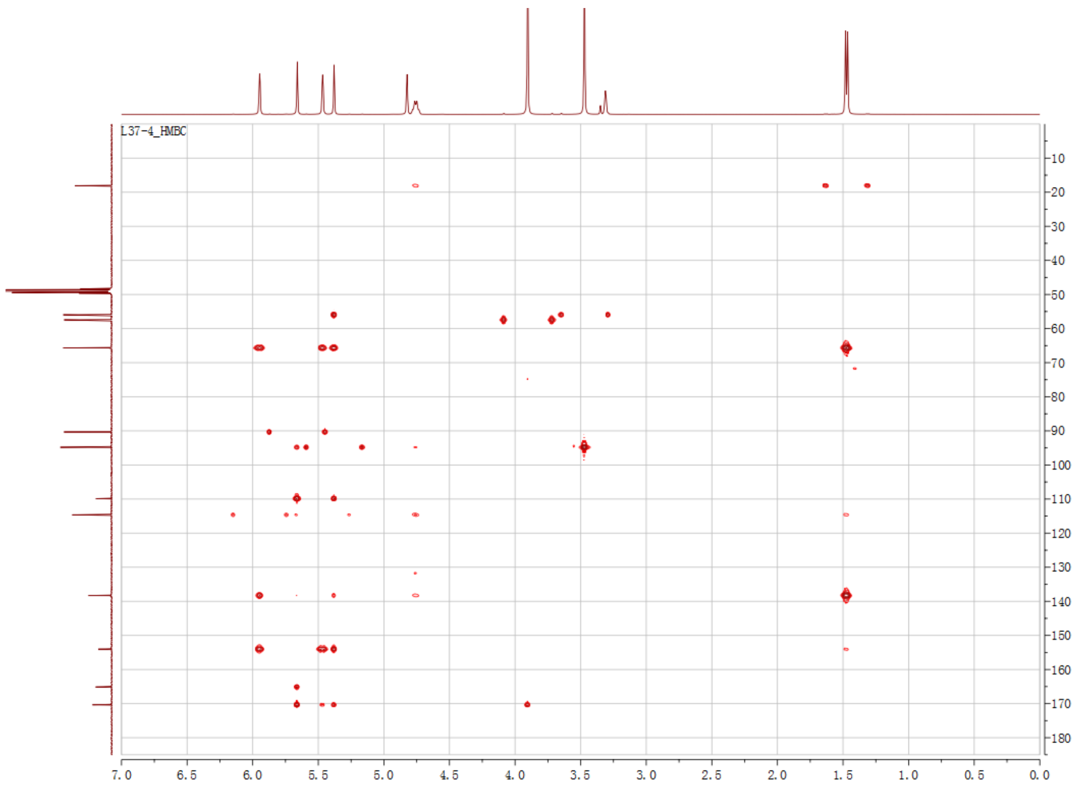

HMBC spectrum of **1** (CD_3OD).

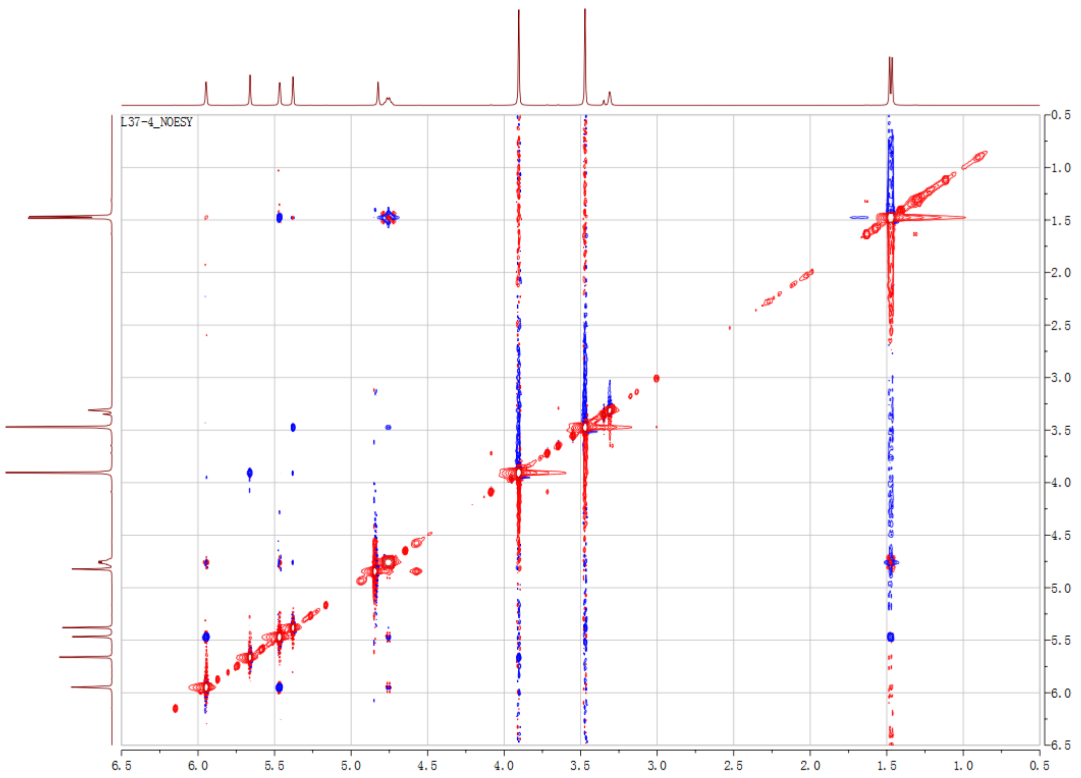

NOESY spectrum of **1** (CD_3OD).

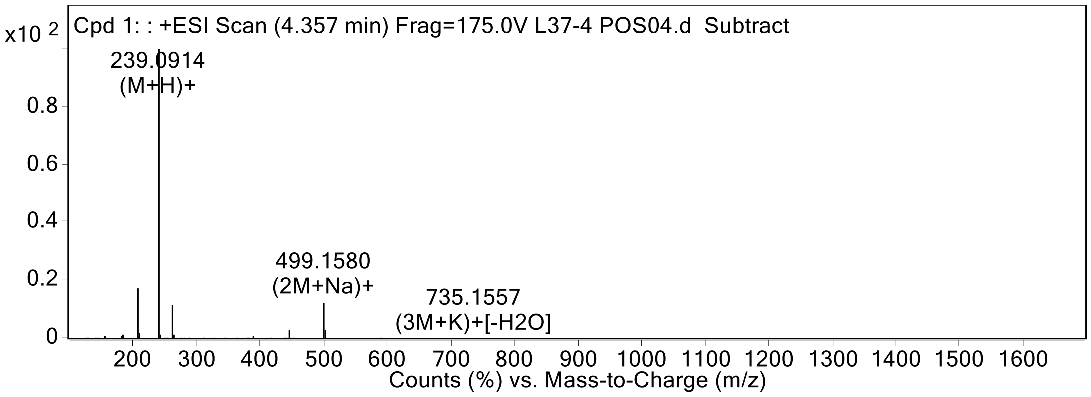

MS Spectrum Peak List

m/z	*Calc m/z*	Diff(ppm)	z	Abund	Formula	Ion
239.0914	239.0914	0.04	1	988227.8	C12H15O5	$(M+H)^+$
499.1580	499.1575	1.04	1	124132.2	C24H28NaO10	$(2M+Na)^+$

HRESIMS spectrum of **1**.

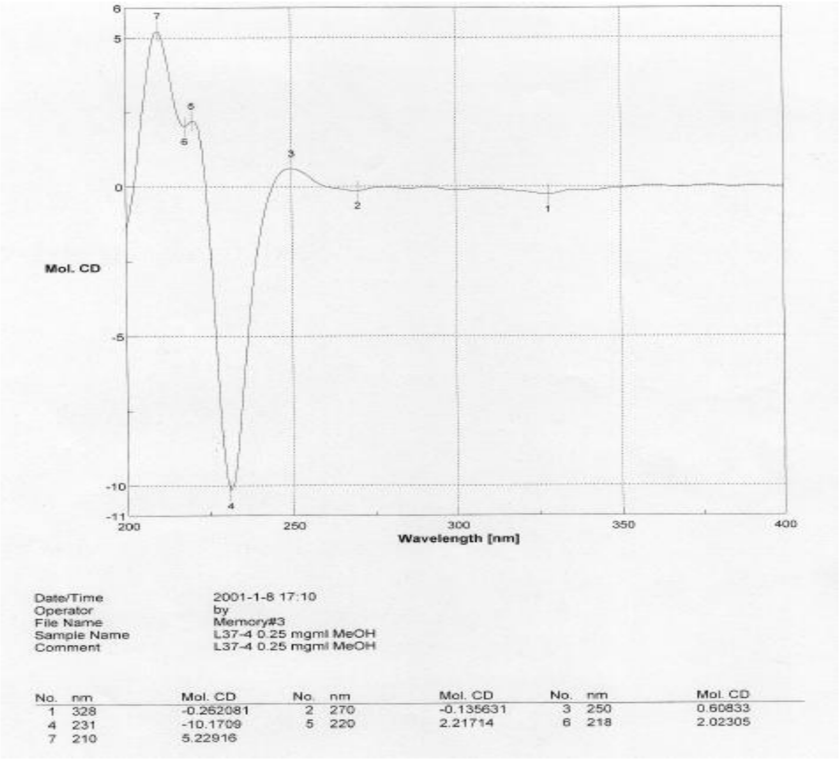

CD spectrum of **1** (CH_3OH).

NMR, HRESIMS, and CD spectra of 2.

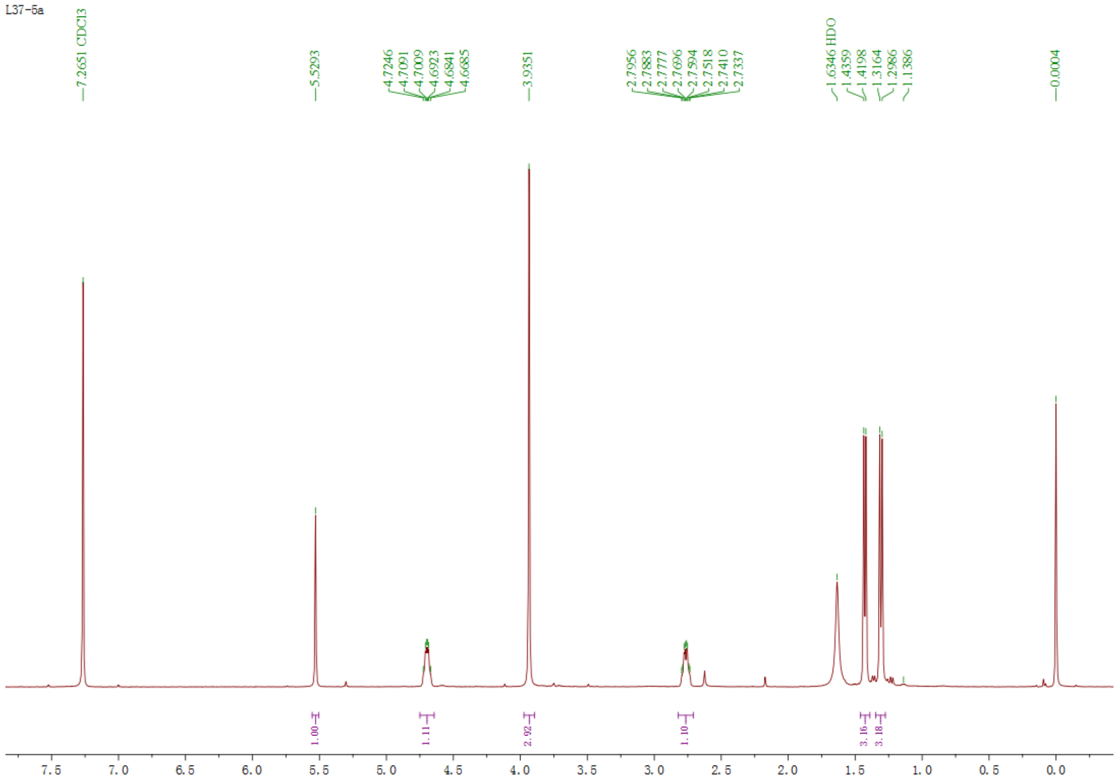

^{1}H NMR spectrum of **2** ($CDCl_3$, 400MHz).

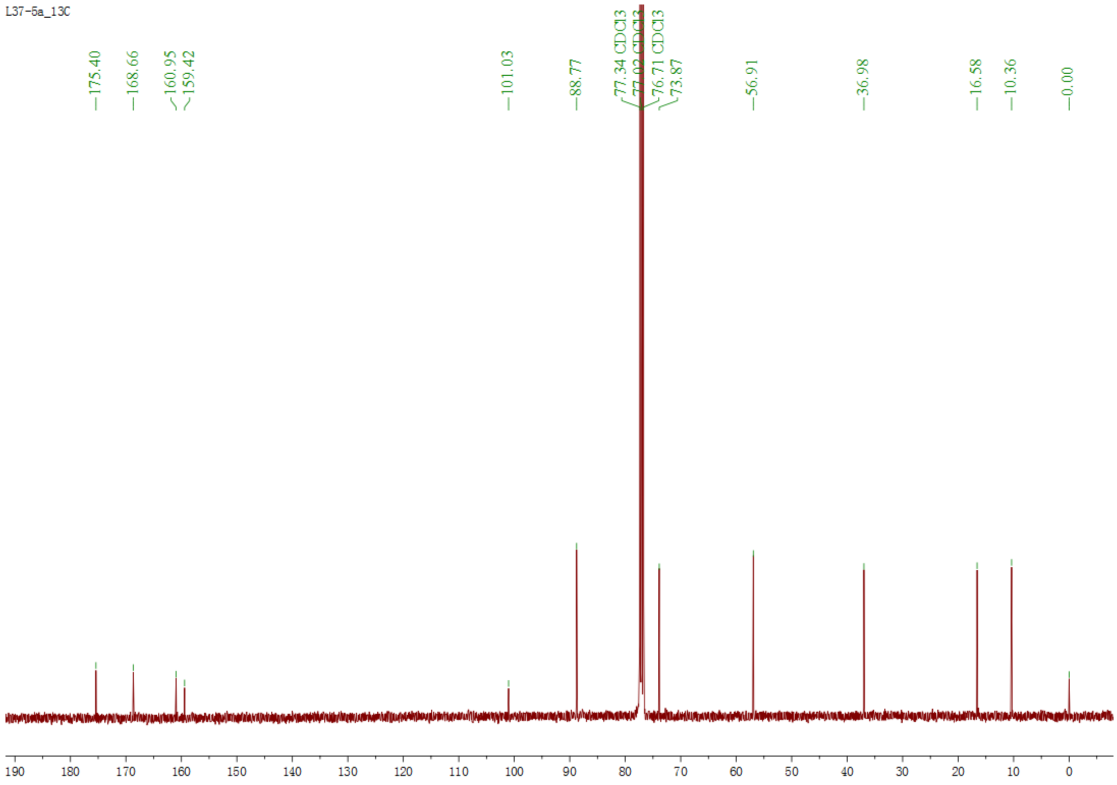

^{13}C NMR spectrum of **2** ($CDCl_3$, 100MHz).

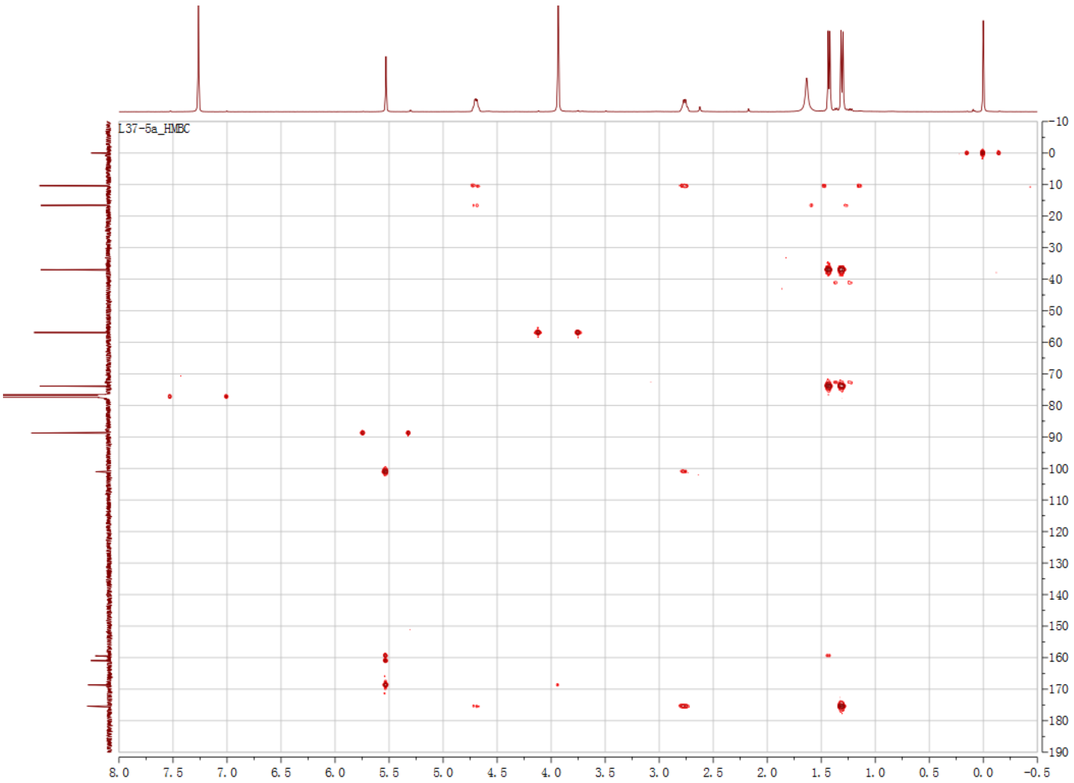

HMBC spectrum of **2** ($CDCl_3$).

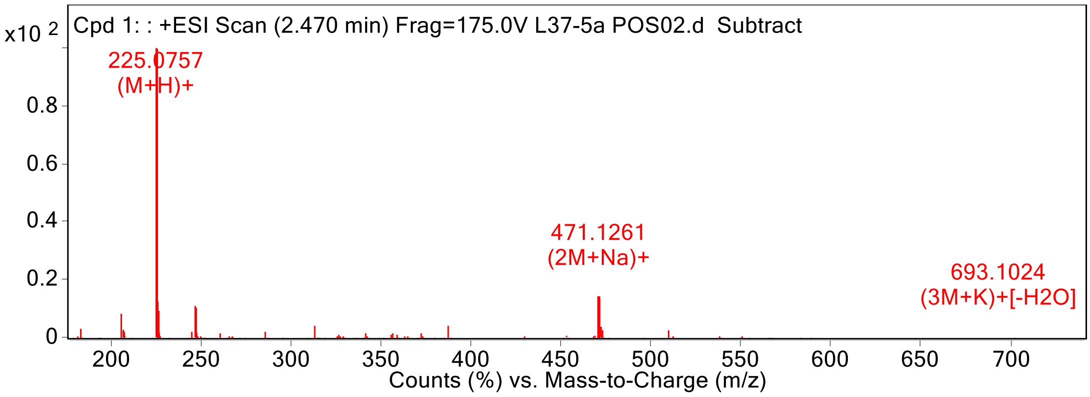

MS Spectrum Peak List

m/z	*Calc m/z*	**Diff(ppm)**	**z**	**Abund**	**Formula**	**Ion**
225.0757	225.0757	-0.28	1	714381.8	C11H13O5	(M+H)+
247.0578	247.0577	0.4	1	77244.8	C11H12NaO5	(M+Na)+
471.1261	471.1262	-0.19	1	98268.8	C22H24NaO10	(2M+Na)+

HRESIMS spectrum of **2**.

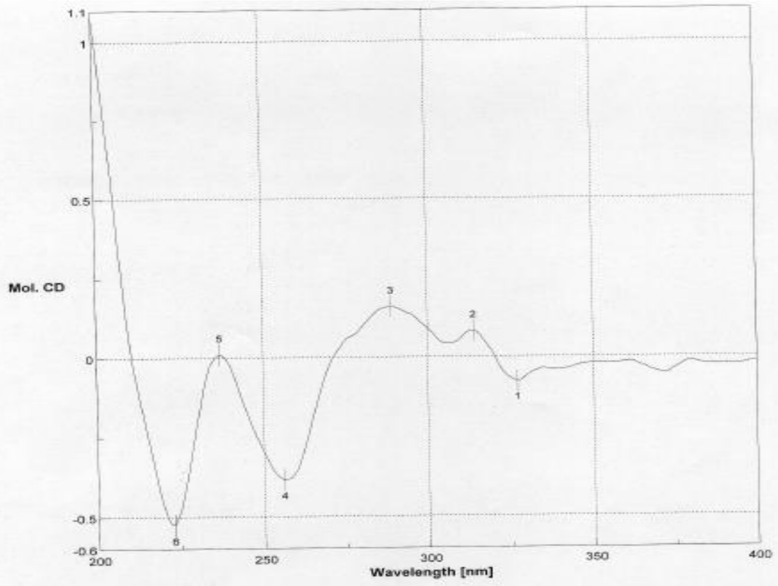

Date/Time	2001-1-8 17:33
Operator	by
File Name	Memory#3
Sample Name	I37-5a 0.25 mg/ml MeOH
Comment	I37-5a 0.25 mg/ml MeOH

No.	nm	Mol. CD	No.	nm	Mol. CD	No.	nm	Mol. CD
1	327	-0.0770443	2	314	0.0810932	3	289	0.158938
4	256	-0.383917	5	237	0.0102058	6	223	-0.524079

CD spectrum of **2** (CH_3OH).

NMR, HRESIMS, and CD spectra of 3.

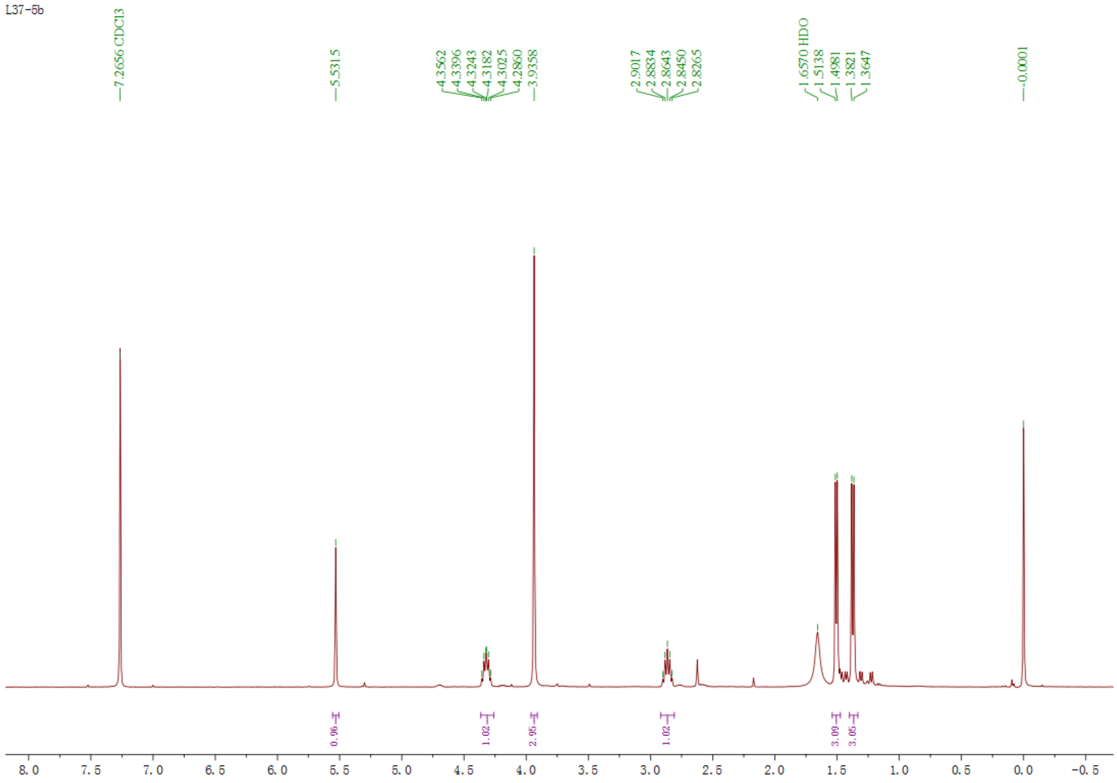

^{1}H NMR spectrum of **3** ($CDCl_3$, 400MHz).

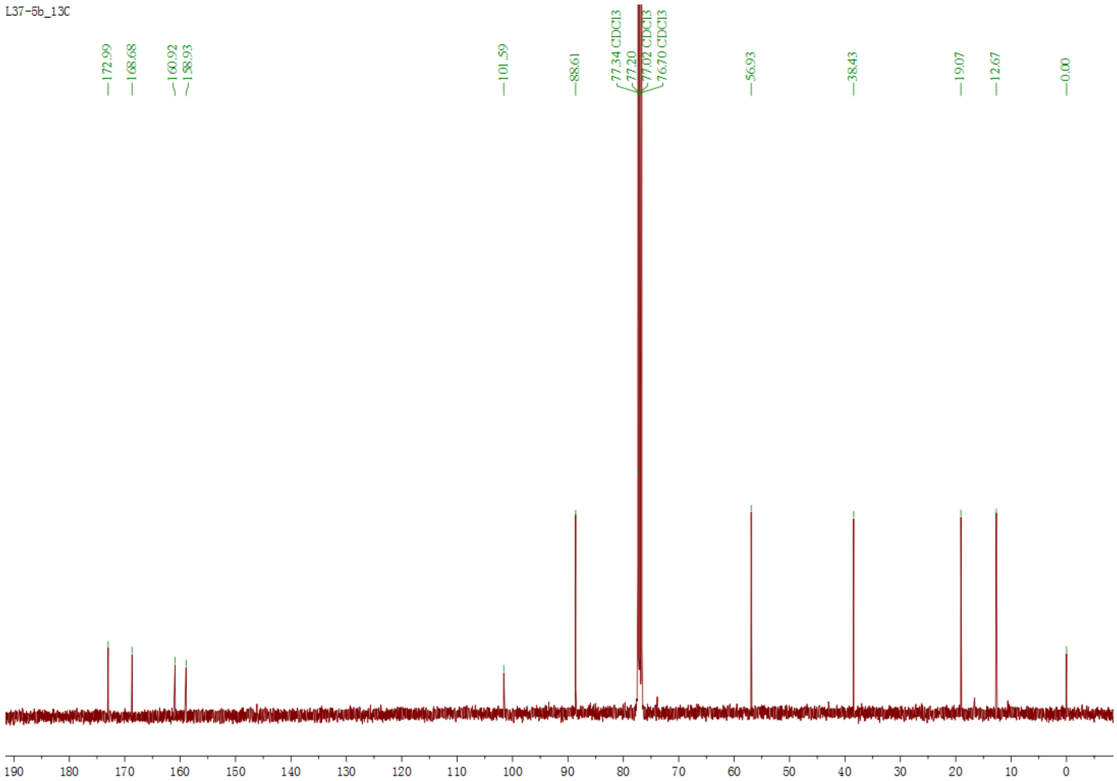

^{13}C NMR spectrum of **3** ($CDCl_3$, 100MHz).

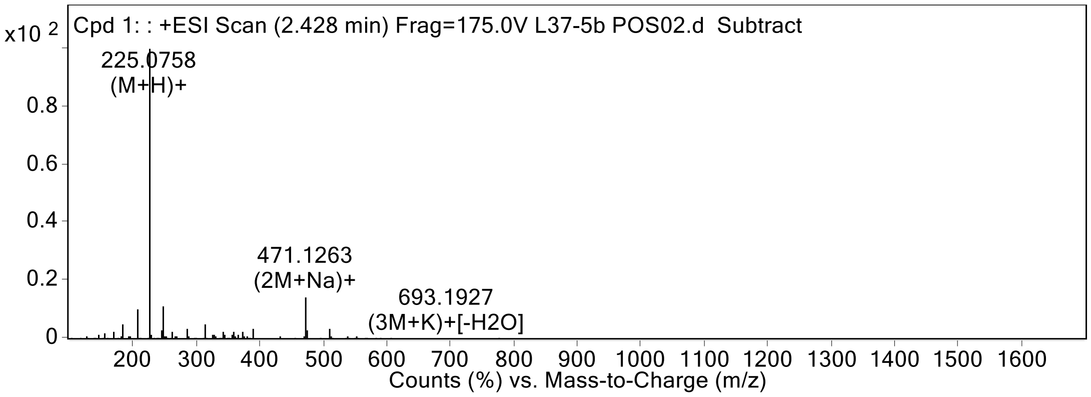

MS Spectrum Peak List

m/z	*Calc m/z*	**Diff(ppm)**	**z**	**Abund**	**Formula**	**Ion**
225.0758	225.0757	0.28	1	768202.1	C11H13O5	(M+H)+
247.0579	247.0577	0.96	1	86776.8	C11H12NaO5	(M+Na)+
471.1263	471.1262	0.38	1	111611.3	C22H24NaO10	(2M+Na)+

HRESIMS spectrum of **3.**

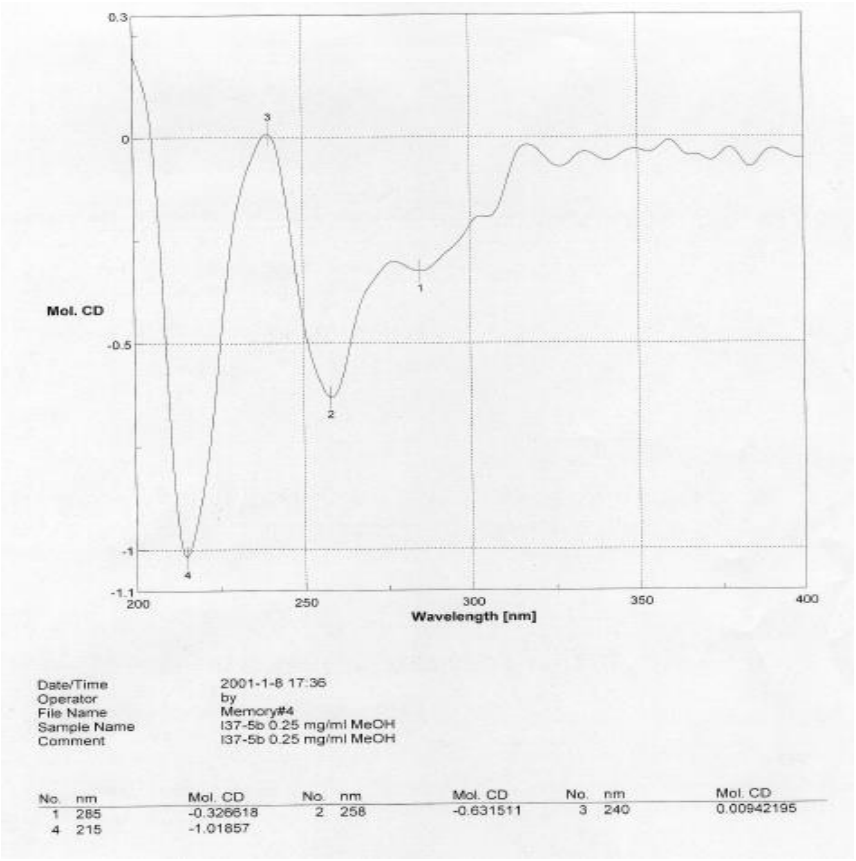

CD spectrum of **3** (CH_3OH).

NMR, HRESIMS, and CD spectra of 4.

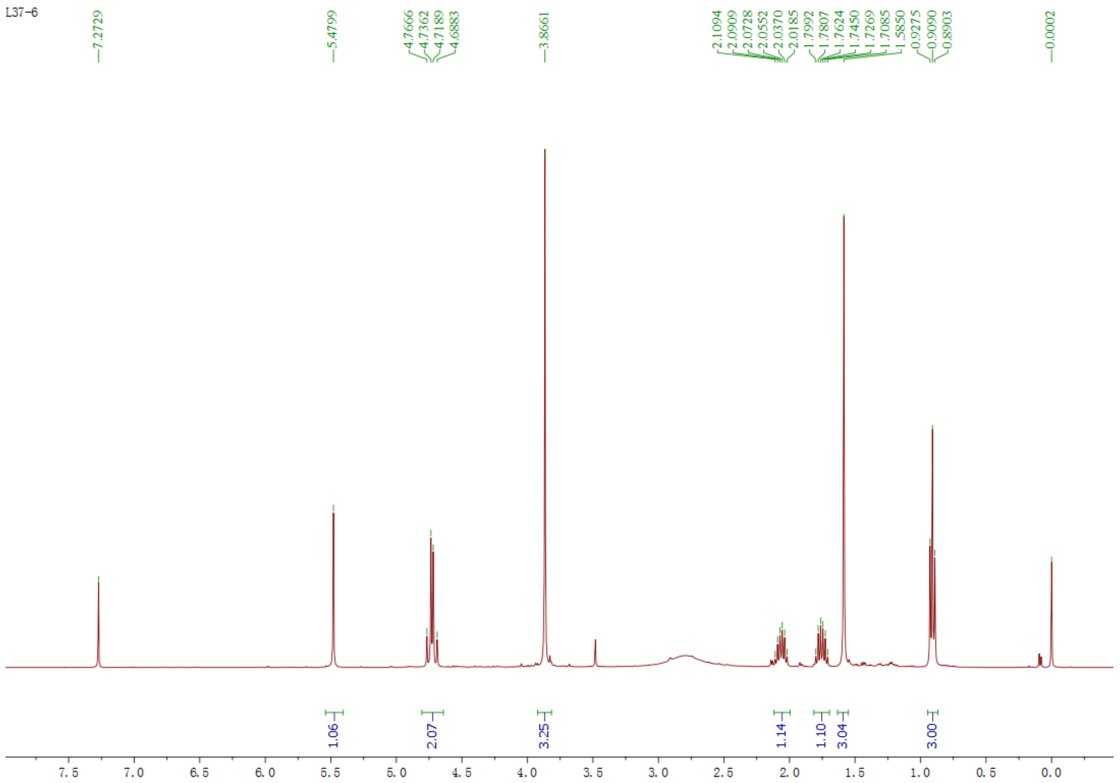

^{1}H NMR spectrum of **4** ($CDCl_3$, 400MHz).

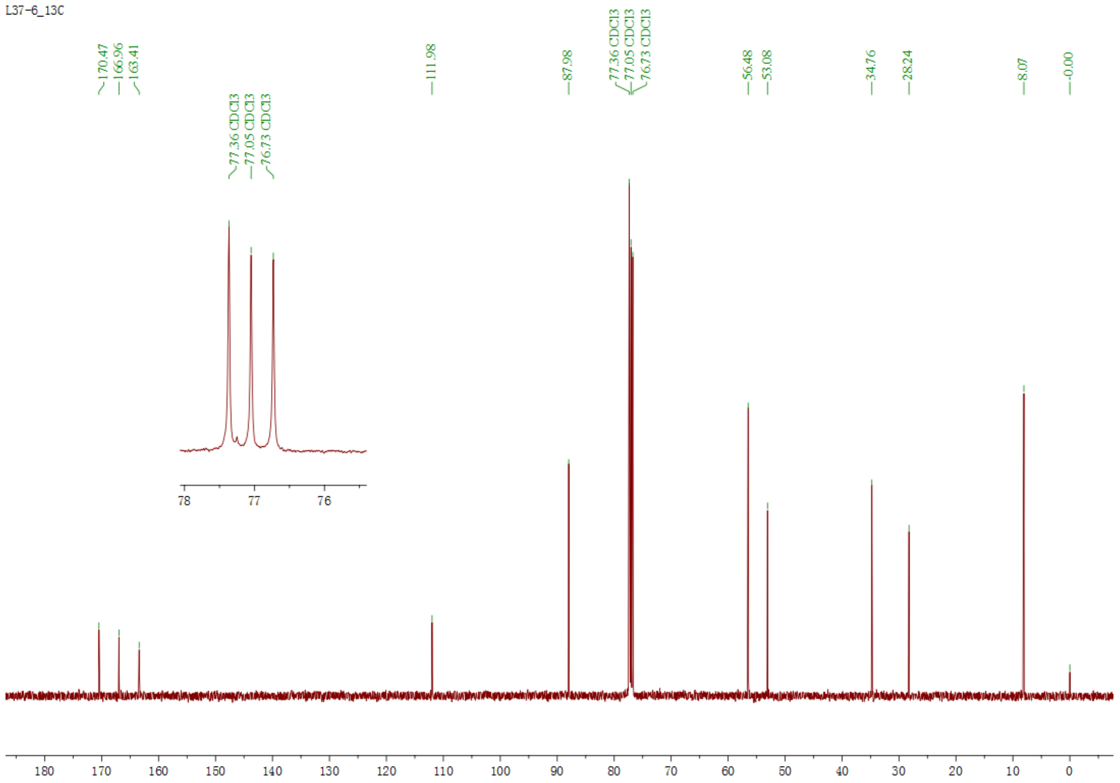

^{13}C NMR spectrum of **4** ($CDCl_3$, 100MHz).

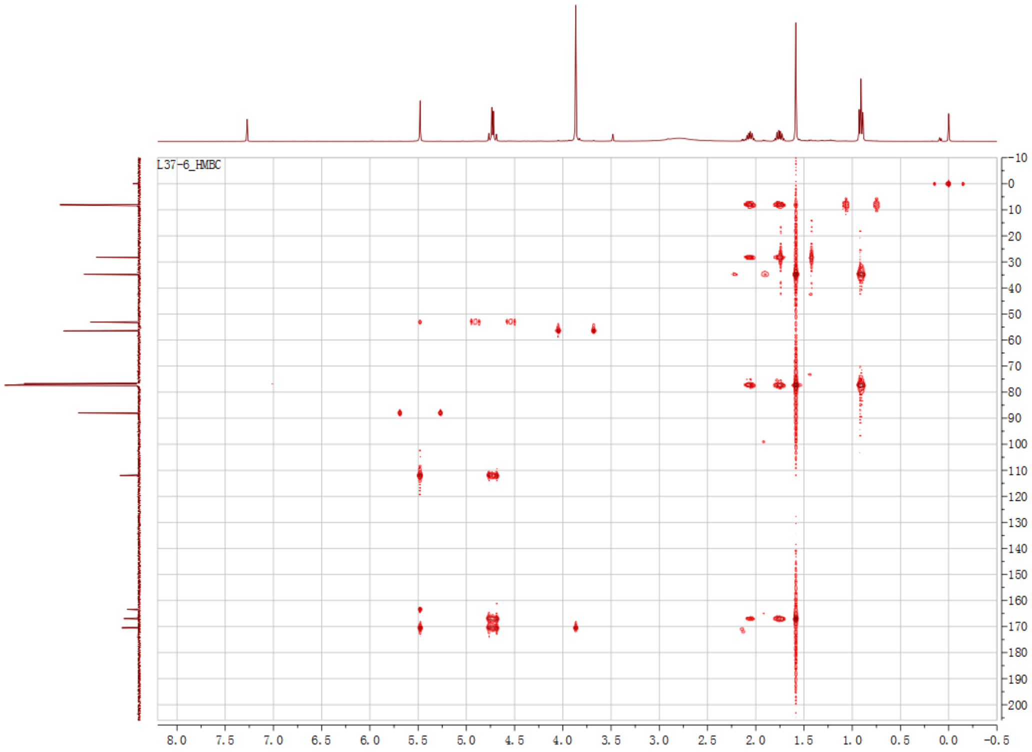

HMBC spectrum of **4** ($CDCl_3$).

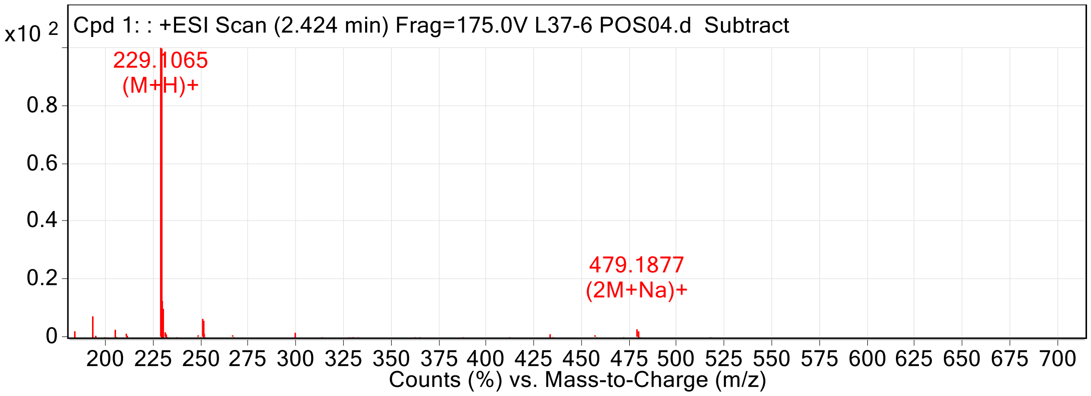

MS Spectrum Peak List

m/z	*Calc m/z*	Diff(ppm)	z	Abund	Formula	Ion
229.1065	229.1071	-2.57	1	651402.5	C11H17O5	(M+H)+
251.0885	251.089	-2	1	41712.8	C11H16NaO5	(M+Na)+
479.1877	479.1888	-2.27	1	15476.9	C22H32NaO10	(2M+Na)+

HRESIMS spectrum of **4** ($CDCl_3$).

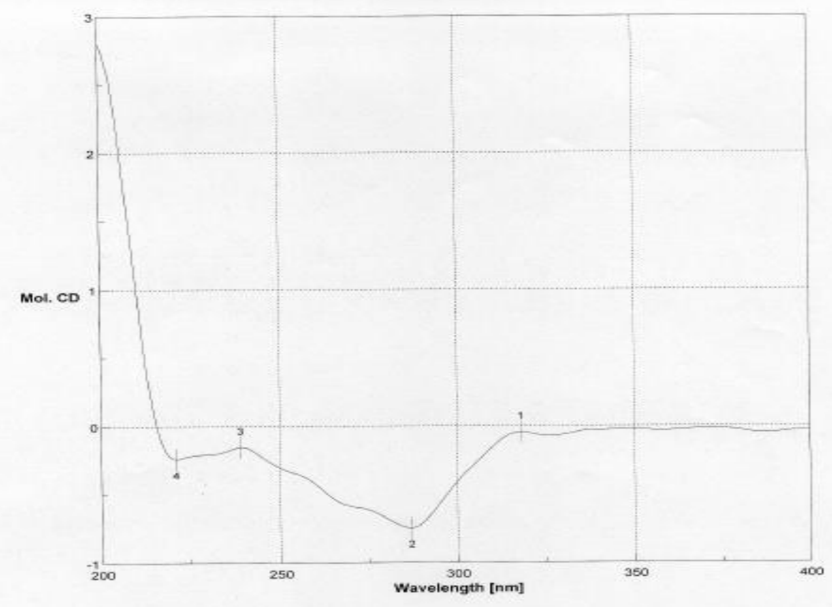

Date/Time	2001-1-8 17:14
Operator	
File Name	Memory#2
Sample Name	L37-6 0.25 mgml MeOH
Comment	

No.	nm	Mol. CD	No.	nm	Mol. CD	No.	nm	Mol. CD
1	318	-0.0476865	2	287	-0.748226	3	239	-0.152577
4	221	-0.238105						

CD spectrum of **4** (CH_3OH).

NMR, HRESIMS, and CD spectra of 5.

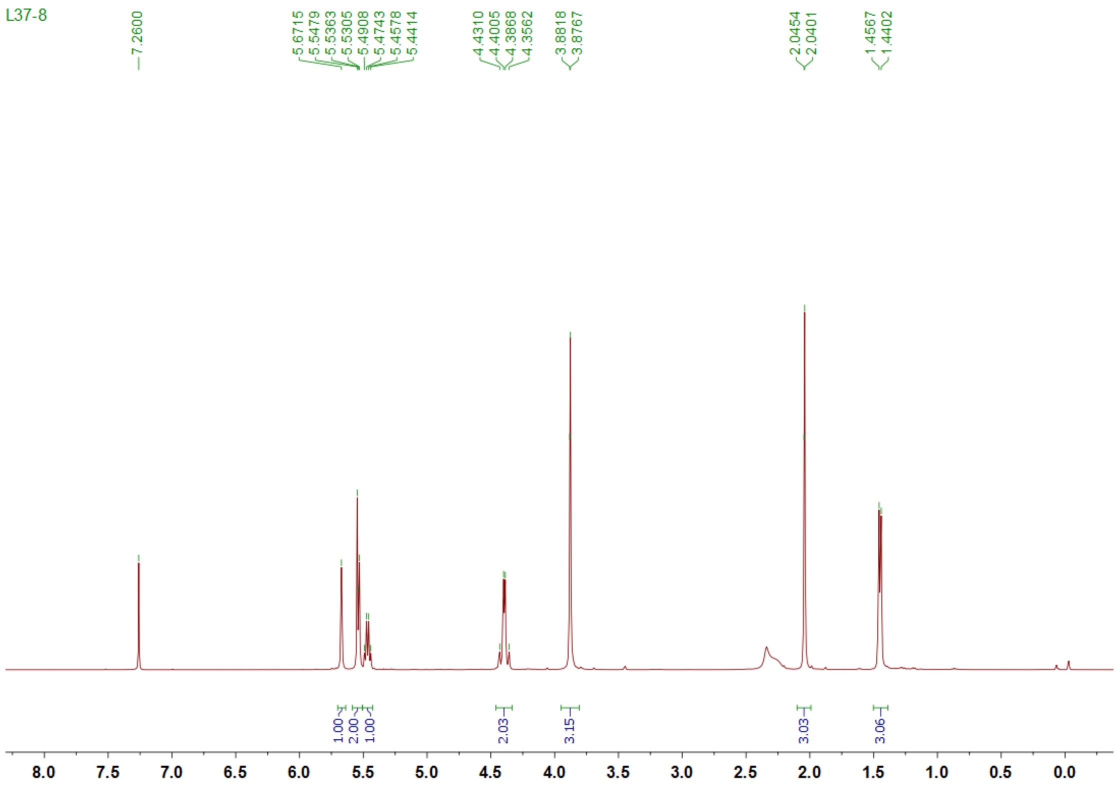

^{1}H NMR spectrum of **5** ($CDCl_3$, 400MHz).

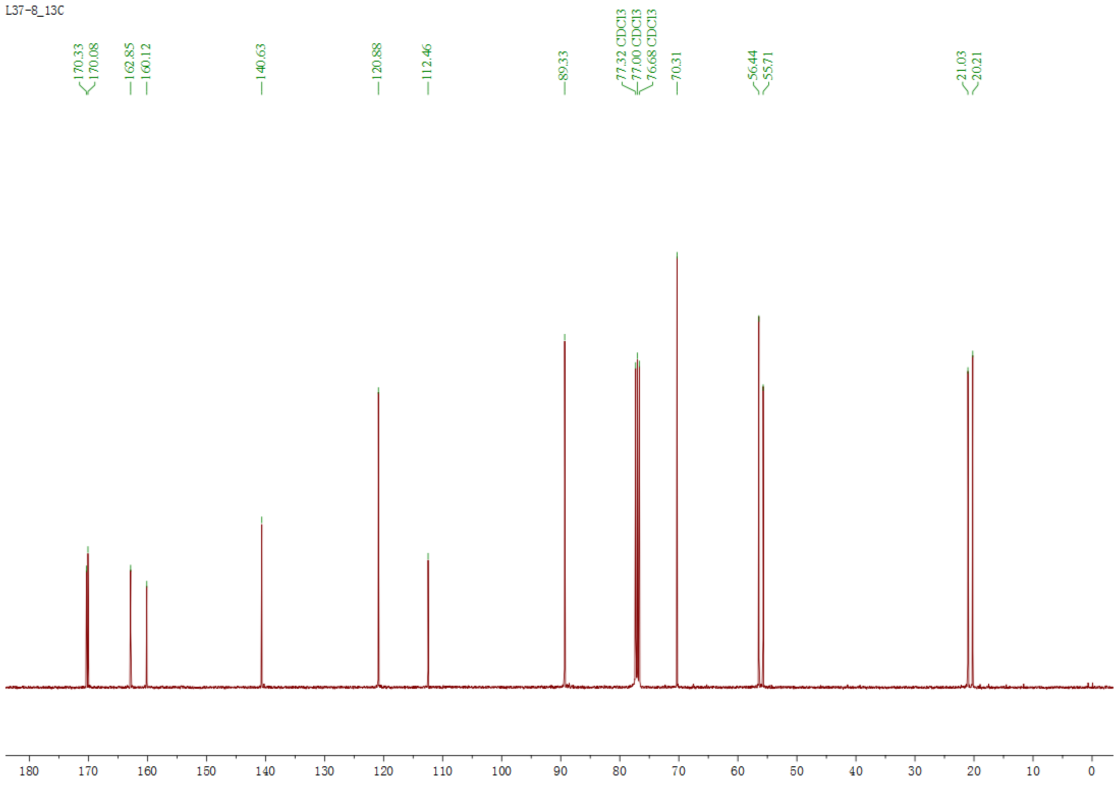

^{13}C NMR spectrum of **5** ($CDCl_3$, 100MHz).

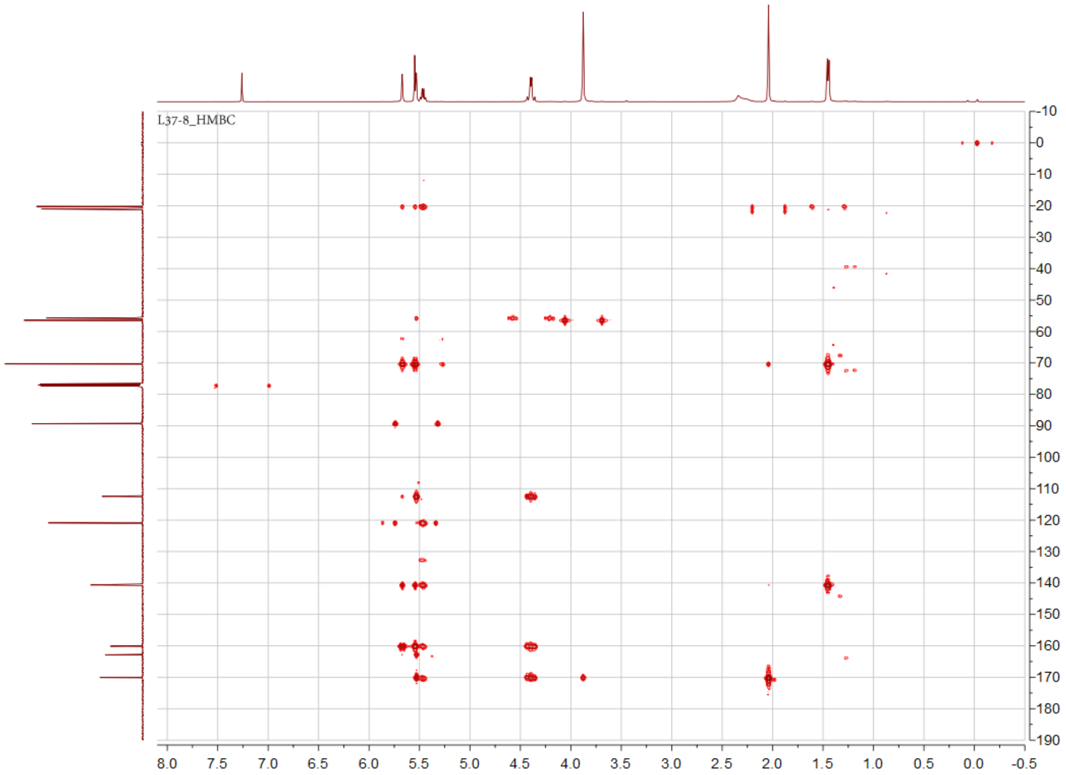

HMBC spectrum of **5** ($CDCl_3$).

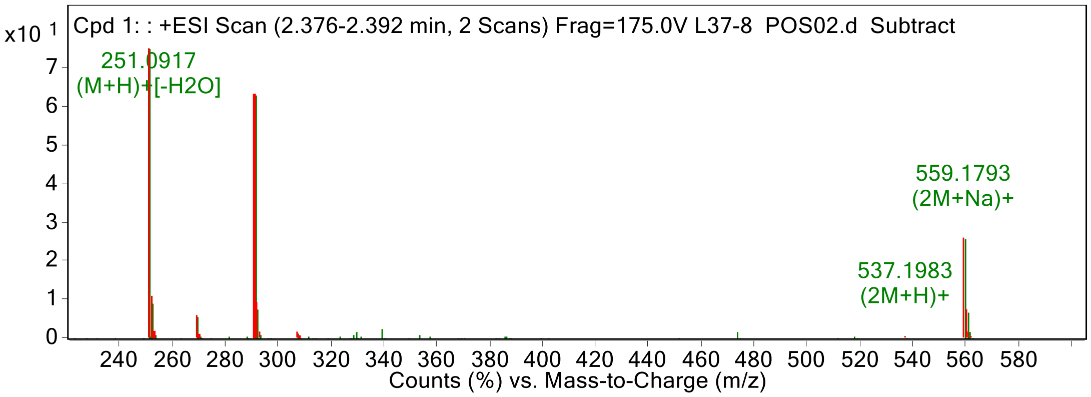

MS Spectrum Peak List

m/z	*Calc m/z*	**Diff(ppm)**	**z**	**Abund**	**Formula**	**Ion**
251.0917	251.0914	1.16	1	412711.1	C13H15O5	(M+H)+[-H2O]
269.1025	269.102	1.93	1	31363.3	C13H17O6	(M+H)+
291.0842	291.0839	1.01	1	346023.3	C13H16NaO6	(M+Na)+
537.1983	537.1967	3.01	1	2765.8	C26H33O12	(2M+H)+
559.1793	559.1786	1.28	1	141551.9	C26H32NaO12	(2M+Na)+

HRESIMS spectrum of **5**.

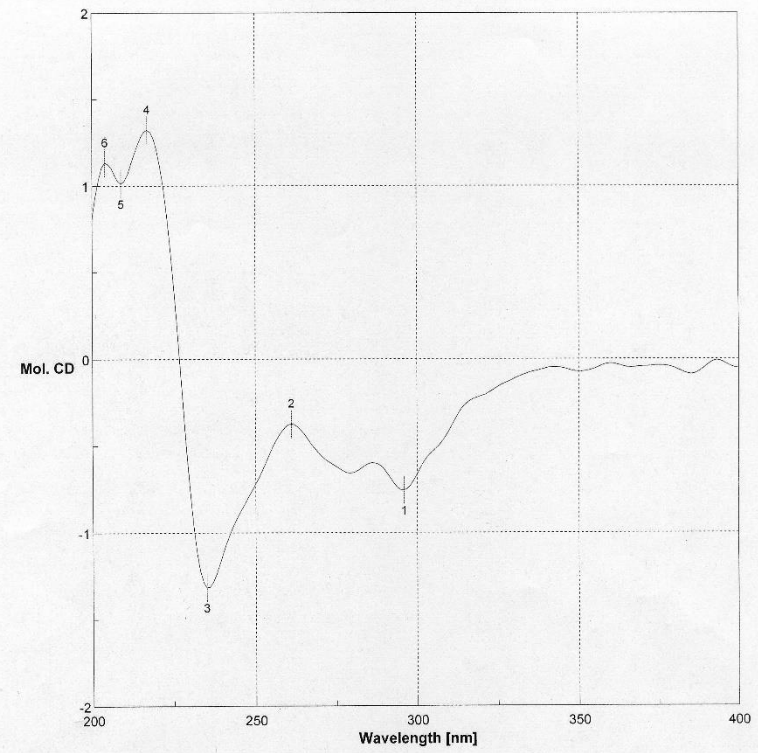

Date/Time	2001-1-8 17:17
Operator	
File Name	Memory#2
Sample Name	L37-8 0.25 mgml MeOH
Comment	

No.	nm	Mol. CD	No.	nm	Mol. CD	No.	nm	Mol. CD
1	296	-0.755601	2	261	-0.373736	3	235	-1.31544
4	217	1.32334	5	209	1.01318	6	204	1.13427

CD spectrum of **5** (MeOH).
